# Thermal [2+2] Cycloaddition Reactions of Perfluorobicyclo[2.2.0]hex-1(4)-ene with Ethylene, Benzene and Styrene: A MEDT Perspective

**DOI:** 10.3390/ma18245675

**Published:** 2025-12-17

**Authors:** Agnieszka Kącka-Zych, Luis R. Domingo

**Affiliations:** 1 Cracow University of Technology, Faculty of Chemical Engineering and Technology, Department of Organic Chemistry and Technology, Warszawska 24, 31-155 Cracow, Poland; 2 Independent Researcher, Avd. Tirso de Molina 20, 46015 Valencia, Spain

**Keywords:** benzene, [2+2] cycloaddition reaction, RIAE analysis, Molecular Electron Density Theory, mechanism

## Abstract

**Highlights:**

**What are the main findings?**

- Thermal [2+2] cycloaddition reactions of perfluorobicyclo[2.2.0]hex-1(4)-ene with ethylene, benzene, and styrene have been researched.
- Thermal [2+2] cycloaddition reactions of bicyclo[2.2.0]hex-1(4)-ene with ethylene, benzene, and styrene have been analyzed.
- Thermal [2+2] cycloadditions proceed through a stepwise mechanism.

**What is the implication of the main finding?**

- RIAE analysis shows that the non-polar transition states are electronically destabilized.
- RIAE analysis shows that the polar transition states cause a strong electronic stabilization.
- The presence of the eight fluorines notably increases the electrophilic character of the bicyclo[2.2.0]hex-1(4)-ene.
- ELF analysis of TSs indicates that forming of the first bond has begun, while the forming of the second bond has not begun.
- The analysis of the geometrical parameters of the TS structures and intermediates portrays a great similarity between them.

**Abstract:**

Thermal [2+2] cycloaddition (22CA) reactions of perfluorobicyclo[2.2.0]hex-1(4)-ene (PFBHE) and bicyclo[2.2.0]hex-1(4)-ene (BHE) with ethylene, benzene and styrene were investigated through the Molecular Electron Density Theory (MEDT) at the UM06-2X/6-311G(d,p) level in benzene. Scrutiny of the DFT-based reactivity indices indicates that the presence of the eight fluorines in PFBHE notably expands the electrophilic nature of this species, participating in polar reactions. These 22CAs proceed through a stepwise mechanism, while the non-polar 22CA reaction of BHE with ethylene requires high energy around 26.6 kcal·mol^−1^, the polar 22CA reaction of PFBHE with styrene requires a low activation energy of 13.2 kcal·mol^−1^. The polar 22CA reaction of PFBHE with benzene presents the highest activation energy, 28.3 kcal·mol^−1^, because of the loss of its aromatic character. Scrutiny of the electron localization function (ELF) at the TSs associated with the first step points that the creation of the C1–C3 bond set about, while that at the TSs associated with the ring-closure means that the creation of the C2–C4 bond has not started yet. At the end, a Relative Interacting Atomic Energy (RIAE) study of these thermal 22CA processes shows that while at the non-polar **TS1a-I** both interacting frameworks are electronically destabilized, at the polar **TS1a-IV**, the hefty global electron density transfer (GEDT) goes ahead towards PFBHE, causing a strong electronic stabilization of this framework, markedly reducing the RIAE activation energy.

## 1. Introduction

Cycloaddition reactions, which enable the creation of arbitrary compounds with high stereo and regioisomeric qualities, are one of the most important groups of organic reactions [1,2]. Cycloaddition reactions are used to obtain small compounds such as cyclopropane or multi-membered cyclic compounds. Cycloadditions are classified using a formula [m+n]. M and n mean the number of unsaturated centers taking part in the reaction. The most common cycloaddition reaction is the [4+2] cycloaddition (42CA) reaction, known as the Diels–Alder reaction [3], which involves the reaction of one 1,3-butadiene and an ethylene derivative [4,5].

**Scheme 1:**
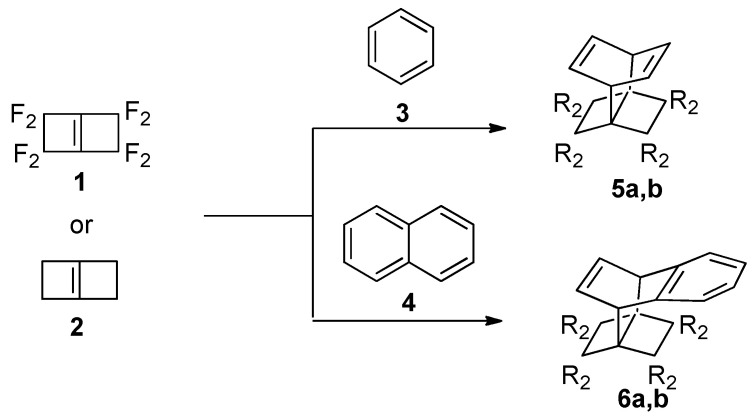
42CA reactions of PFBHE **1** and BCHE **2** with benzene **3** and naphthalene **4**.

The 42CA process of perfluorobicyclo[2.2.0]hex-1(4)-ene (PFBHE, **1**) and bicyclo[2.2.0]hex-1(4)-ene (BHE, **2**) with benzene **3** and naphthalene **4** were recently scrutinized within Molecular Electron Density Theory (MEDT) [6] at the MPWB1K/6-311G(d,p) theory level (see Scheme 1) [7,8]. These 42CA reactions take place through a synchronous and non-concerted one-step mechanism that requires moderate energy. However, PFBHE **1** can also participate in [2+2] cycloaddition (22CA) reactions towards ethylenes derivatives. There are many examples of reactions in which two competing [4+2] and [2+2] pathways are feasible [9,10,11,12,13].

**Scheme 2:**
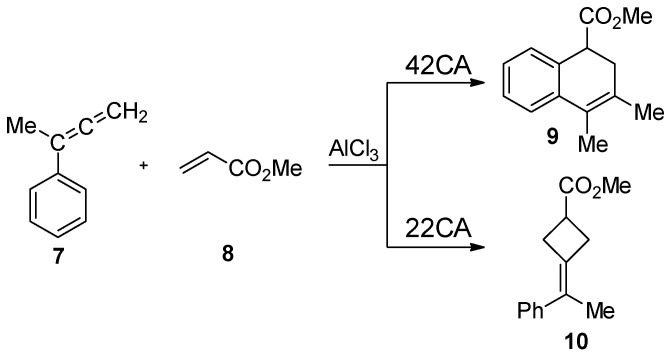
Two possible [4+2] and [2+2] reaction directions in the cycloaddition of 1-methyl-1-phenylallene **7** and methylacrylate **8**.

One example of a reaction in which the 42CA and 22CA reactions are feasible is the reaction between 1-methyl-1-phenylallene **7** and methylacrylate **8** (Scheme 2) [9]. In this case, both cycloaddition reaction paths are possible; the asynchronicity of the 42CA reaction shifts the character of the mechanism from one-step to a stepwise for the 22CA reaction, escalating the polar nature of these Lewis acid (LA) catalyzed cycloaddition reactions.

**Scheme 3:**
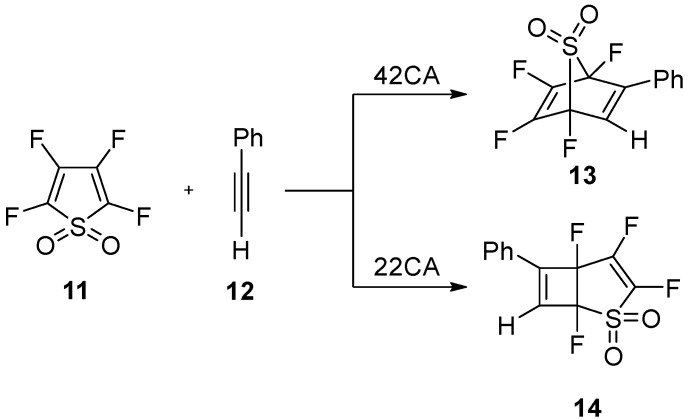
Two possible [4+2] and [2+2] reaction paths in reaction tetrafluorothiophene-S,S-dioxide **11** with styrene **12**.

Also, in the reaction of tetrafluorothiophene-S,S-dioxide **11** with terminal acetylenes as styrene **12,** two possible reaction paths giving the [4+2] and [2+2] cycloadducts were considered (Scheme 3) [10]. In this reaction, the [4+2] reaction path proceeded in a synchronous fashion, while the [2+2] one was considered forbidden, yet the 22CA reaction can dominate the course of the reaction.

**Scheme 4:**
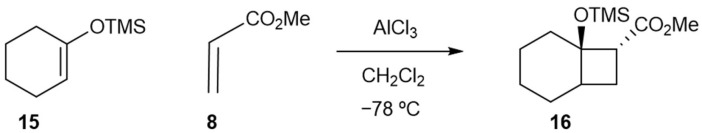
LA promoted the 22CA reaction of silyl enol ether **15** with methylacrylate **8**.

The LA induced 22CAs of silyl enol ether **15** with the α,β-unsaturated esters conduct into the cyclobutanes with a high degree of trans stereoselectivity (see Scheme 4) [14]. A theoretical analysis of the silyl enol ether **15** with methyl acrylate **8** showed that without LA, the 42CA occurs through asynchronous bond-formation reactions, with a high activation enthalpy of 21.3 kcal·mol^−1^ [15]. Coordination of the AlCl_3_ LA to the carbonyl oxygen of methyl acrylate **8** contributes to replacing the mechanism with a two-step mechanism with the formation of a zwitterionic intermediate [15].

Recently, an energy decomposition analysis based on the Interacting Quantum Atoms (IQA) [16], namely the Relative Interacting Atomic Energy (RIAE) [17], was deployed within MEDT.

IQA is grounded on the quantum theory of Atoms-in-Molecules (AIM) [18,19], permits the division of the total energy calculated through Density Functional Theory (DFT) [20] into intra- and interatomic energy components on the grounds of the Kohn-Sham (KS) [21,22] analysis.

**Scheme 5:**
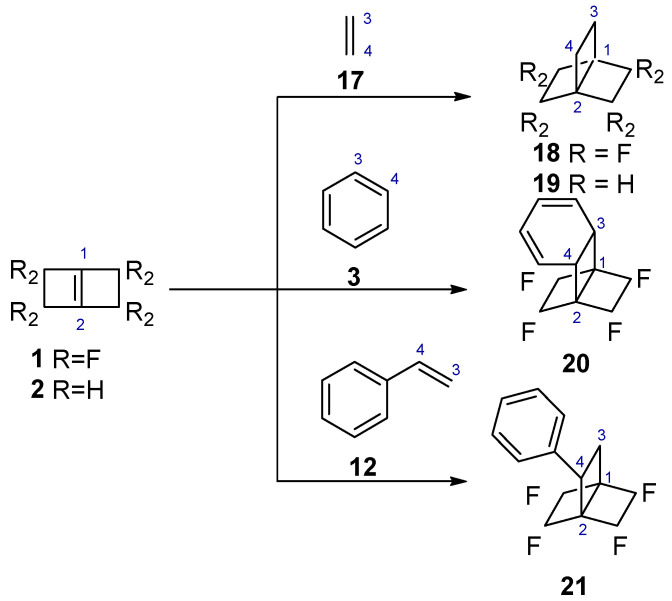
22CA reactions of BHE **2** with and those of PFBHE **1** with ethylene **17**, benzene **3**, and styrene **12**.

As mentioned, the cycloaddition chemistry of the 42CA reactions of PFBHE **1** and BCHE **2** with benzene **3** and with naphthalene **4** was recently studied (see Scheme 1) [8]. However, a possible competitive 22CA reaction should be theoretically considered. Herein, a MEDT research of the 22CA of BCHE **2** with ethylene **17**, and those of PFBHE **1** with ethylene **17**, benzene **3**, and styrene **12** is performed at the UM06-2X/6-311G(d,p) level (Scheme 5). An RIAE analysis of the TSs responsible for the kinetics of these 22CAs is performed to analyze the electronic factors responsible. The end of this MEDT study is to characterize, both energetically and electronically, these thermal 22CA reactions.

## 2. Computational Details

The DFT calculations in this article were conducted using the M06-2X functional [23], together with the 6-311G(d,p) [24] basis set. Detailed calculation procedures are provided in the Supplementary Material.

## 3. Results and Discussion

This study has been shared in five different parts: (i) first, an analysis of the electronic structure at the ground state (GS) of reagents are conducted; (ii) next, an analysis of the DFT-based reactivity indices for reagents is carried out; (iii) in this point, the potential energy surfaces (PESs) of the 22CAs of PFBHE **1** and BHE **2** with ethylene **17**, benzene **3** and styrene **12** were analyzed; (iv) next, the ELF topological analysis of the stationary points concerned in the studied 22CAs is performed; and finally, (v) a RIAE analysis of the TSs associated with the 22CAs of BHE **2** with ethylene **17**, and those of PFBHE **1** with ethylene **17** and styrene **12** is performed to determine the electronic factors responsible for the activation barriers.

### 3.1. ELF and NPA Characterization of Reagents

**Figure 1:**
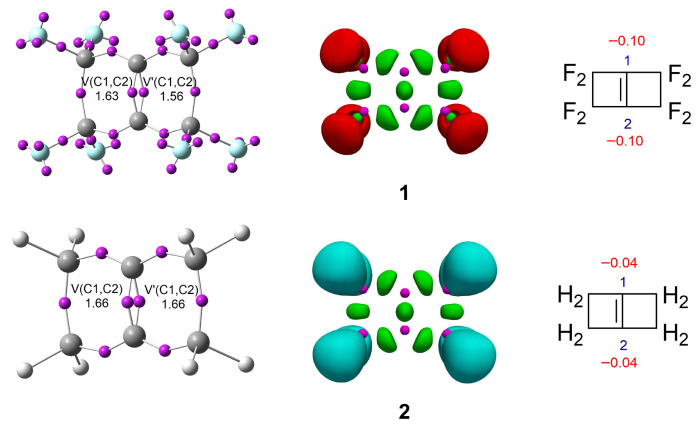
The ELF study of PFBHE **1** and BHE **2**, together with the NPA atomic charges and proposed Lewis-like structures.

**Figure 2:**
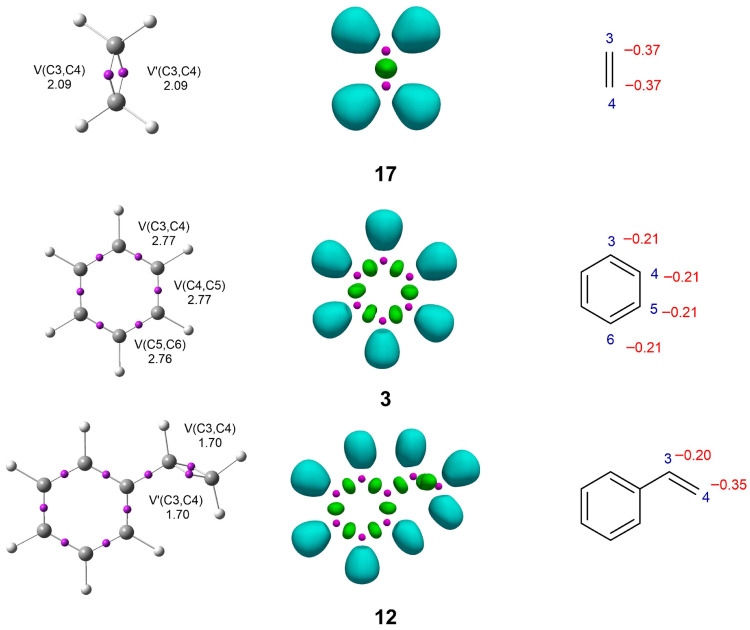
The ELF analysis of ethylene **17**, benzene **3**, and styrene **12** together with the NPA atomic charges and proposed Lewis-like structures.

At the beginning, we decided to determine the structure of the reagents. We carried out the ELF [25,26,27] analysis, which allows us to check the electron density distribution [28]. The most relevant ELF valence attractor positions, the natural atomic charges, and the offered Lewis-like structures for PFBHE **1**, BHE **2**, ethylene **17**, benzene **3**, and styrene **12**, are given in Figure 1 and Figure 2.

The ELF topology of PFBHE **1** indicates the appearance of two V(C1,C2) and V’(C1,C2) disynaptic basins having a population of 1.63 e and 1.56 e, respectively. These disynaptic basins are relevant to the appearance of a C1–C2 double bond. A close behavior is noticed in the ELF of the BHE **2** structure. The distribution of two V(C1,C2) and V’(C1,C2) disynaptic basins, integrating each 1.66 e, indicates the appearance of a C1–C2 double bond (Figure 1). The NPA analysis indicates the presence of negative charges on the C1 and C2 carbon atoms. In PFBHE **1**, both C1 and C2 carbons have a charge of −0.10 e; in the case of BHE **2**, it is slightly smaller and equal to −0.04 e.

The ELF study of ethylene **17** indicates the occurrence of two V(C3,C4) and V’(C3,C4) disynaptic basins, each one integrating 2.09 e (Figure 2). ELF of benzene **3** shows the presence of six equivalent V(Ci,Cj) disynaptic basins, interblend each one ca 2.77 e. Basins population values indicate the presence of partial double bonds in the entire benzene ring. ELF of styrene **12** indicates the appearance of two V(C3,C4) and V’(C3,C4) disynaptic basins, integrating 1.70 e each one, which are relevant to C3–C4 double bond. The NPA analysis [29,30] performances in an important negative charge on the two C3 and C4 carbon atoms of ethylene **17**, −0.37 e. The six carbons in benzene **3** also exhibit the same negative charge of −0.21 e. The charges are distributed differently in the ethylene region of styrene **12**. The largest negative charge is situated on the terminal C4 carbon of styrene **12**, while the C3 carbon shows a charge of −0.20 e (Figure 2). The negative charges found in all carbons of these hydrocarbon compounds are the result of the carbon atom being more electronegative than the hydrogen atom.

### 3.2. Research on Reactivity Indices of the Reagents

Next, the chemical reactivity of the reagents was analyzed according to the DFT-based reactivity indices [31,32,33,34] for PFBHE **1**, BHE **2**, ethylene **17**, benzene **3**, and styrene **12** at the GS. The results of these calculations are presented in Table 1.

The electronic chemical potential [31] µ of BHE **2**, µ = −2.46 eV is much higher than that of PFBHE **1**, µ = −5.38 eV. In turn, the electronic chemical potential µ of the rest reagents belongs to the range of −3.30–−3.43 eV (Table 1). It is worth noting that in the case of reactions of PFBHE **1** with **15**, **3**, and **12**, the GEDT [31] would proceed from **15**, **3**, and **12** towards PFBHE **1**. However, in the case of reactions between BHE **2** with **15**, **3**, and **12**, it is expected that the GEDT would proceed from BHE **2** toward these species.

According to the absolute scale [32] of the electrophilicity [33] ω index, PFBHE **1**, ω = 2.08 eV, is classified as a strong electrophile, styrene **12**, ω = 1.13 eV and benzene **3**, ω = 0.80 eV, as moderate electrophiles, and BHE **2**, ω = 0.41 eV, and ethylene **17**, ω = 0.73 eV, as a marginal electrophile. Thus, it is expected that only PFBHE **1** will act as an electrophilic species.

According to the absolute scale [32] of the nucleophilicity [34] N index, styrene **12**, N = 3.09 eV, and BHE **2**, N = 2.94 eV, are categorized as strong nucleophiles, benzene **3**, N = 2.49 eV, as moderate nucleophile, and ethylene **17**, N = 1.87 eV, and PFBHE **1**, N = 0.26 eV, as marginal nucleophiles. Consequently, neither ethylene **17** nor PFBHE **1** will participate in polar reactions as nucleophiles.

The local changes in electron density on the electrophile and nucleophile along a polar reaction can be noticed by the analysis of the electrophilic $P_{k}^{+}$ and nucleophilic $P_{k}^{-}$ Parr functions [35], which are shown in Table 2 together with the local electrophilicity ω_k_ and nucleophilicity *N_k_* values [32].

A study of electrophilic $P_{k}^{+}$ Parr function of PFBHE **1** points out that the most electrophilic centers in this molecule are located on C1 and C2 carbons. Both centers show the same value of the electrophilic $P_{k}^{+}$ Parr function, $P_{k}^{+}$ = 0.48. In the case of the BHE **2**, C1 and C2 carbons show the same values of electrophilic $P_{k}^{+}$ and nucleophilic $P_{k}^{-}$ Parr functions. Interesting, in spite of the high electrophilic $P_{k}^{+}$ Parr function at C1 and C2, $P_{k}^{+}$ = 0.49, the local electrophilicity is very low, ω_k_ = 0.20 eV. On the other hand, the nucleophilic $P_{k}^{-}$ Parr function at C1 and C2, $P_{k}^{+}$ = 0.45, demonstrating the strong nucleophilic character at these centers, *N_k_* = 1.33 eV (Table 2). Finally, in styrene **12**, the most electrophilic $P_{k}^{+}$ and nucleophilic $P_{k}^{-}$ Parr functions are located on the C4 carbon ($P_{k}^{+}$ = 0.47 and $P_{k}^{-}$ = 0.50), but only this carbon is nucleophilically activated, *N_k_* = 1.54 eV.

### 3.3. Study of the 22CA Reactions of PFBHE ***1*** and BHE ***2*** with Ethylene ***17***, and PFBHE ***1*** with Benzene ***3*** and Styrene ***12***

This section has been shared in two subsections: (i) study of the 22CA reactions of PFBHE **1** and BHE **2** with ethylene **17**; and (ii) analysis of the 22CA reactions of PFBHE **1** with benzene **3** and with styrene **12**.

#### 3.3.1. Analysis of the 22CA of PFBHE **1** and BHE **2** with Ethylene **17**

**Scheme 6:**
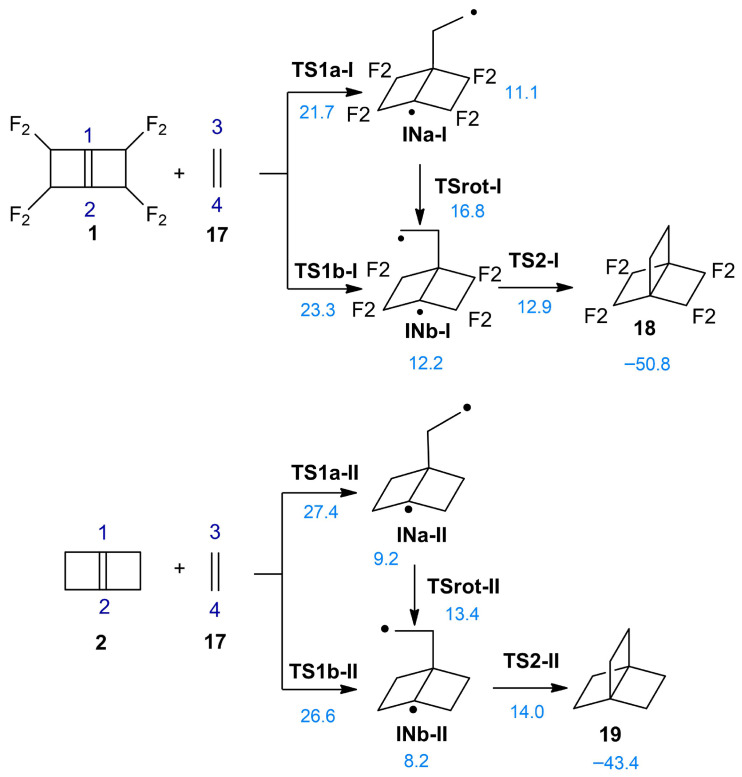
22CA reaction of PFBHE **1** and BHE **2** with ethylene **17** together with UM06-2X/6-311G(d,p) relative energies, in kcal·mol^−1^ (in blue).

First, the PES of the 22CA of PFBHE **1** and BHE **2** with ethylene **17** was explored (see Scheme 6). These 22CA reactions occur through a stepwise mechanism, with the creation of two intermediates. Along with the making of the C1–C3 single bond, two stereoisomeric reaction paths are feasible, a and b, yielding two conformational intermediates, which are interconverted via a C1–C3 single bond rotation. These reaction paths are characterized by the different approaches of the methylene C3 carbon of ethylene **17** to the bridged C1 carbon of PFBHE **1** and BHE **2**, and they are characterized by the C2–C1–C3–C4 dihedral angles, ca. 180 (a) and 77 (b) degrees. The relative energies of the TSs, intermediates, and products are given in Scheme 6; also, the total energies are placed in Table S1 in the Supplementary Material.

According to calculations (Scheme 6), we can conclude that: (i) the activation energies relevant to creation of the first C1–C3 single bond are 21.7 (**TS1a-I**), 23.3 (**TS1b-I**), 27.4 (**TS1a-II**), and 26.6 (**TS1b-II**) kcal·mol^−1^. Consequently, the activation energies associated with the 22CAs of PFBHE **1** are ca. 6 and 3 kcal·mol^−1^ lower that those connected with the 22CA reaction of BHE **2**; (ii) for the 22CA reaction of PFBHE **1**, the reaction path a is kinetically preferred by 1.6 kcal·mol^−1^, while for the 22CA of BHE **2** is the reaction path b by 0.7 kcal·mol^−1^. Both reaction paths are competitive in the two 22CA reactions; (iii) formation of the intermediates is endothermic by 11.1 (**INa-I**) 12.9 (**INb-I**) 9.2 (**INa-II**) and 8.2 (**INb-II**) kcal·mol^−1^; an amount of almost 10 (PFBHE **1**) and 18 (BHE **2**) kcal·mol^−1^ are realized after passing the first TSs; (iv) conversion of the intermediates **INa-I** and **INa-II** in **INb-I** and **INb-II** via **TSrot-I** and **TSrot-II** has a barrier of 5.7 and 4.2 kcal·mol^−1^; (v) from **INb-I** and **INb-II**, the activation energies correlative with the formation of the second C2–C4 single bond are 0.03 and 5.8 kcal·mol^−1^, respectively; and finally, (vi) formation of the [2+2] cycloadducts **18** and **19** are strongly exothermic by −50.8 and −43.4 kcal·mol^−1^, respectively. Consequently, the presence of the eight fluorines in PFBHE **1** has a relevant influence in both the kinetic and thermodynamics of these 22CA reaction.

**Figure 3:**
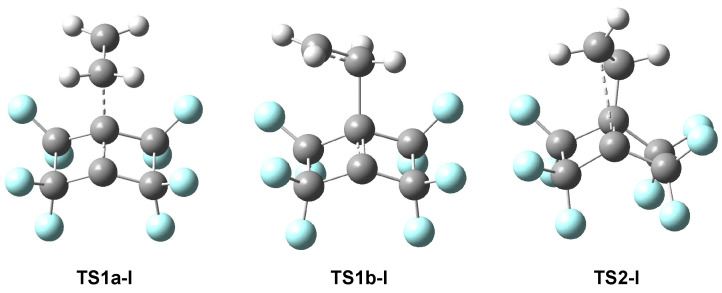
UM06-2X/6-311G(d,p) geometries in benzene of the **TS1a-I**, **TS1b-I** and **TS2-I** involved in the 22CA of PFBHE **1** with ethylene **17**. The geometries of **INa-I** and **INb-I** are very similar to those of **TS1a-I** and **TS1b-I**.

The geometries of the TSs participated in the 22CA reaction of PFBHE **1** and ethylene **17** are presented in Figure 3, also the key geometrical parameters of stationary points participated in the two 22CA reactions, together with the GEDT at the TSs and intermediates are given in Table 3. An analysis of the Cx–Cy distances at the TSs and intermediates connected with the two 22CA reactions shows a great similitude. The distances betwixt the C1 and C3 carbons are 1.829 (**TS1a-I**), 1.846 (**TS1a-II**), 1.908 (**TS1a-II**) and 1.893 (**TS1b-II**) Å, while the C2–C4 distances are greater than 3.4 Å. The short C1–C3 distances at the four TSs connected with the first steps, lower than 1.91 Å, indicate that they are very advanced. The C-C bond is created with length in the range 2.0–1.9 Å [31]. The distance between C1–C3 informs us that this bond will be created later at the four TSs. At the intermediates the lengths of the new C1–C3 single bonds are in the range 1.52–1.53 Å, while the C2–C4 distances remain greater than 3.1 Å. Finally, at **TS2-I** and **TS2-II**, the distances between the C2 and C4 carbons are 2.693 and 2.485 Å, respectively, indicating that the creation of the second C2–C4 bond has not yet begun.

Scrutiny of the GEDT [31] at the TS involved in the first step of these 22CAs allows for quantifying the polar nature of the influence [31]. GEDT merits higher than 0.05 e classify the reactions as polar, while GEDT values lower than 0.05 e classify them as non-polar. The GEDT at **TS1a-I** and **TS1b-I** are ca 0.15 e, while the GEDT at **TS1a-II** and **TS1b-II** are less than 0.03 e. Thus, while the 22CA reaction of PFBHE **1** is classified as polar, that of BHE **2** is classified as non-polar. These GEDT values account for the lower activation energies for the polar 22CA reaction of PFBHE **1** than that of BHE **2** (see later). This change in the GEDT is a result of the powerful electrophilic nature of PFBHE **1**, ω = 2.08 e, resulting from the presence of the eight fluorines. Note that the electrophilicity of BHE **2** is 0.73 eV (see Table 1).

Recently, a useful and unequivocal classification of the organic process derivative from the way of the stream of the electron density at the polar TSs was established [36]. Polar processes are categorized as forward electron density flux (FEDT) when the electron density fluxes from the reference molecule to the other one, as the reverse electron density flux (REDF) when the flux goes in the reverse direction [36]. Non-polar reaction with GEDT ≤ |0.05| e are categorized as null electron density flux (NEDF) [36]. Thus, while the 22CA reaction of PFBHE **1** with ethylene **17** is classified as REDF, that of BHE **2** with ethylene **17** is categorized as NEDF.

The GEDT found at **TS1a-II**, 0.01 e, indicates the non-polar nature of this 22CA, a similar value to that computed at the non-polar Diels-Alder process among 1,3-butadiene **22** and ethylene **17** [31]. On the other hand, the activation energy connected with this 22CA, 27.4 kcal·mol^−1^, is even lower than that related to the stepwise reaction path connected with the DA process of 1,3-butadiene **22** and ethylene **17** listed at the UM06-2X/6-311G(d,p) in benzene, 35.4 kcal·mol^−1^. These behaviors reject the assumption made in 1965 by Hoffmann, in which 42CA reactions are thermally allowed by symmetry, while the 22CA reactions are forbidden [37].

#### 3.3.2. Analysis of the 22CA of PFBHE **1** with Benzene **3** and with Styrene **12**

**Scheme 7:**
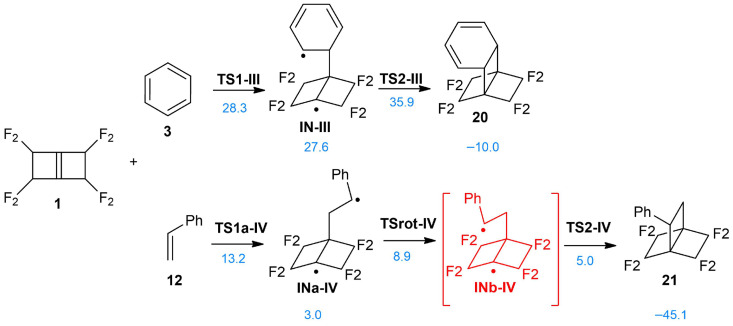
22CA reaction of PFBHE **1** with benzene **3** and styrene **12** together with UM06-2X/6-311G(d,p) relative energies, in kcal·mol^−1^ with regard to the independent reagents.

Next, the PESs of the 22CA reactions of PFBHE **1** with benzene **3** and with styrene **12** were explored. The analyzed 32CA reactions undergo via different mechanisms (see Scheme 7); the 22CA reaction of PFBHE **1** with benzene **3** undergoes via a two-step mechanism including the creation of **IN-III**, while the 22CA reactions of PFBHE **1** with styrene **12** goes ahead via a stepwise routine with the initial creation of the **INa-IV**, which by a further C1–C3 bond rotation yield the intermediate **INb-IV**. The further ring-closure in this intermediate provides the final [2+2] cycloadduct **21** (see Scheme 7). After passing **TSrot-IV**, the search for **INb-IV** as a stationary point was unfeasible, because all optimizations of a feasible **INb-IV** yield directly without a barrier the [2+2] cycloadduct **21**. The relative energies of all the stages are presented in Scheme 7, as well as in Table S2 in the Supplementary Material.

According to calculations (Scheme 7), we can conclude that: (i) the activation energies connected with the creation of the first C1–C3 bond are 28.3 (**TS1-III**) and 13.2 (**TS1-IV**) kcal·mol^−1^. The activation energy relevant the 22CA with styrene **12** experiences a strong reduction; (ii) while creation of **IN-III** is strongly endothermic by 27.6 kcal·mol^−1^, creation of **IN-III** is slightly endothermic by only 3.0 kcal·mol^−1^; (iii) the energy of the reaction is connected generally with creation of the second C2–C4 single bond are 8.2 (**TS2-III**) and 2.0 (**TS2-IV**) kcal·mol^−1^; (iv) in the 22CA relate benzene **3**, **TS2-III** is located 7.5 kcal·mol^−1^ above **TS1-III**. Consequently, the second step becomes the rate-determining step of this 22CA reaction; and finally, (v), while formation of the [2+2] cycloadduct **20** is exothermic by 10.0 kcal·mol^−1^, creation of the **21** is strongly exothermic by 45.1 kcal·mol^−1^. The different reactivity of benzene **3** with respect to styrene **12** along the creation of the first C1–C3 single bond is the loss of aromaticity in the former, along with the participation of the aromatic C3 carbon of benzene **3** along the cycloaddition reaction [38]. Note that formation **IN-III** is 24.60 kcal·mol^−1^ higher in energy than that for **INa-IV**. As the 42CA of PFBHE **1** with benzene **3** proceeds with a barrier of 32.3 kcal/mol [8], the corresponding 22CA reaction, which has a barrier of 35.9 kcal·mol^−1^, is non-competitive.

**Figure 4:**
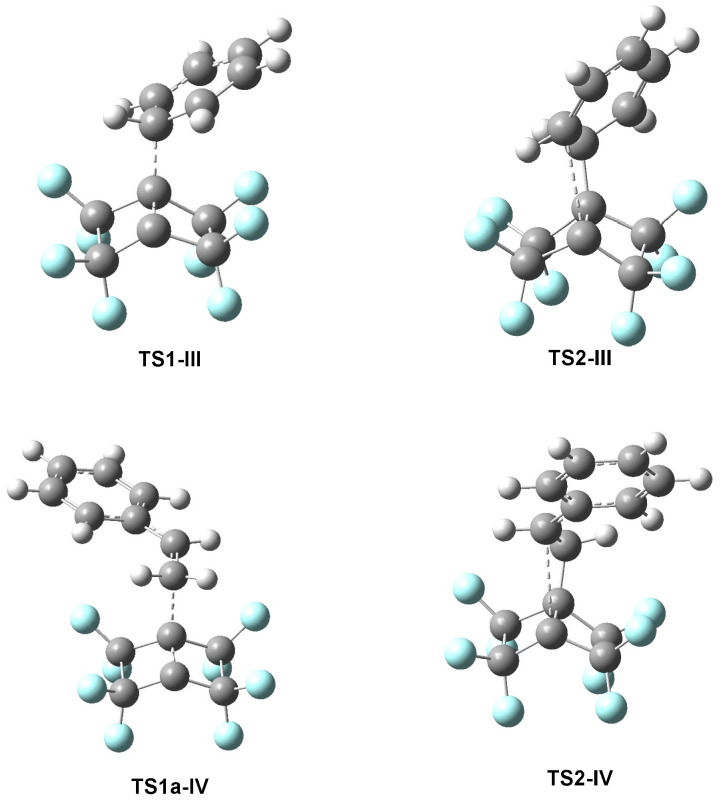
UM06-2X/6-311G(d,p) geometries in benzene of the **TS1-III**, **TS2-III**, **Ts1a-IV**, and **TS2-IV** connected with the 22CA of PFBHE **1** with benzene **3** and styrene **12**.

The optimized geometries of the TSs concerned in the 22CA of PFBHE **1** with benzene **3** or styrene **12** are shown in Figure 4, while the key geometrical parameters of the stationary points concerned in these 22CAs, together with the GEDT at the TSs and INs, are shown in Table 4. A comparative study of the Cx-Cy distances at the TSs and connected with these 22CA reactions indicates that these distances at **TS1-IV** and **TS2-IV** associated with the 22CA reaction with styrene **12** are closely to those at **TS1b-I** and **TS2b-I** related to the 22CA of PFBHE **1** with ethylene **17**, while these distances at **TS1-III** and **TS2-III** connected with the 22CA reaction of benzene **3** are more advanced. Thus, at these TSs, the distances between the influence of the two C-C centers are 1.631 and 2.598 Å, respectively.

Finally, the GEDT at the TS involved in the first step of these 22CA reactions was computed to quantify the polar nature of this process. The GEDT at **TS1-III** and **TS1a-IV** are 0.29 and 0.22 e, respectively. These high values, which are higher than those at **TS1b-I**, 0.15 e, account for the high polar nature of these 22CAs. The GEDT at the most adverse **TS1-III** is higher than that at **TS1a-IV** as a result of the shorter C1–C3 distance.

The GEDT computed at **TS1-III** and at **TS1a-IV** allow for the classification of both 22CA reactions as REDF. Consequently, PFBHE **1** participates as an electrophile in these polar 22CA reactions, in agreement with its high electrophilic character (see Table 1).

### 3.4. ELF Topological Analysis of the TSs and Intermediates Involved in the 22CA Reactions

To get to know electron density shifts along the process paths related to the 22CA reactions of the PFBHE **1** and BHE **2** with ethylene **17**, and those of PFBHE **1** with benzene **3** and styrene **12**, an ELF analysis at the TSs and intermediates was performed.

#### 3.4.1. ELF Analysis of the TSs and Intermediates Concerned in the 22CA Reaction Between PFBHE **1** and Ethylene **17**

**Figure 5:**
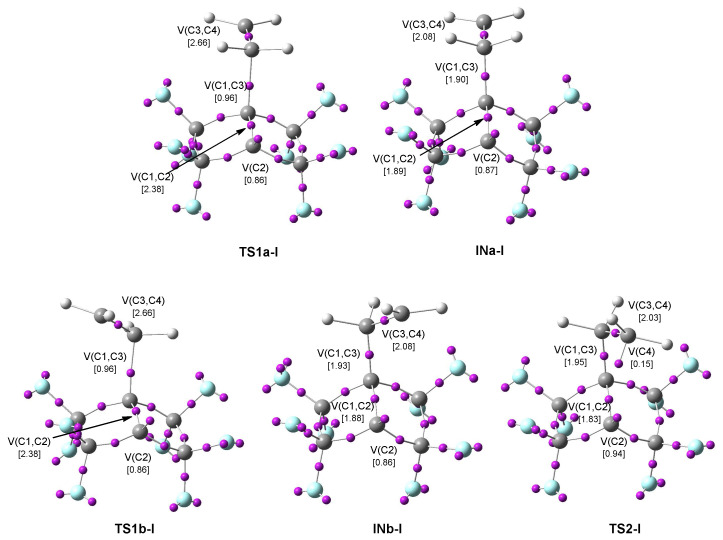
ELF study of TSs and intermediates concerned in the 22CA of the PFBHE **1** and ethylene **17**.

The electronic structures of the TSs and intermediates concerned in the 22CA reactions of PFBHE **1** and BHE **2** with ethylene **17** were explained with the aid of ELF analysis. The results of these analyses are shown in Figure 5.

At **TS1a-I**, d(C1,C3) = 1.829 Å and d(C2,C4) = 3.900 Å, the more relevant valence basins are the V(C1,C2), V(C1,C3) and V(C3,C4) disynaptic basins, integrating each one 2.38 e, 0.96 e and 2.66 e, respectively (Figure 5). The population of these basins indicates that while the C1–C2 and C3–C4 double bond have been depopulated, a new V(C1,C3) disynaptic basin is generated by the creation of the new C1–C3 bond that has begun at this TS. ELF of **TS1a-I** also shows the appearance of a new V(C2) monosynaptic basin (0.83 e) related to a *pseudoradical* C2 center. The ELF picture of **TS1b-I**, d(C1,C3) = 1.846 Å and d(C2,C4) = 3.412 Å, is close to that of **TS1a-I**.

ELF picture of **INa-I**, d(C1,C3) = 1.529 Å and d(C2,C4) = 3.980 Å, shows that while the V(C1,C2) and V(C3,C4) disynaptic basins have been depopulated equated to those of **TS1a-I** by 2.38 e and 0.96 e, respectively, the value of V(C1,C3) disynaptic basin growth to 1.90 e. This behavior indicates that the creation of the new C1–C3 bond has been completed at this intermediate. The ELF picture of **INb-I**, d(C1,C3) = 1.529 Å and d(C2,C4) = 3.101 Å, is very close to that of **INa-I**.

Finally, ELF picture of **TS2-I**, d(C1,C3) = 1.516 Å and d(C2,C4) = 2.693 Å, shows the formation of a new V(C4) monosynaptic basin (0.15 e) related to a *pseudoradical* C4 center. This *pseudoradical* center and the *pseudoradical* C2 center present at **TS1b-I** (see Figure 5) are essential for the creation of the second C2–C4 single bond after passing **TS2-I**.

The present ELF topological analysis indicates that while the making of the C1–C3 bond has already started at **TS1a-I** and **TS1b-I**, the creation of the second C2–C3 bond has not started yet at **TS2-I**.

The present ELF study of the TSs and intermediates indicates that while the creation of the first C1–C3 bond has already begun at **TS1a-I** and **TS1b-I**, the creation of the second C2–C3 bond has not started yet at **TS2-I**.

#### 3.4.2. ELF Study of the TSs and Intermediates Participated in 22CA of the BHE **1** and Ethylene **13**

**Figure 6:**
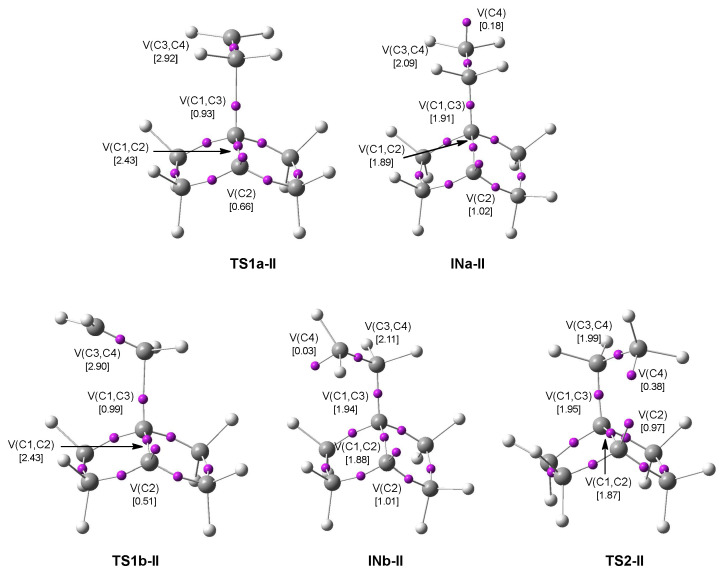
ELF basin attractor positions of the TSs and intermediates taking part in the 22CA of the PFBHE **1** and ethylene **17**.

The ELF results are pictured in Figure 6. ELF picture of **TS1a-II**, d(C1,C3) = 1.908 Å and d(C2,C4) = 3.997 Å, shows the presence of three V(C1,C2), V(C1,C3) and V(C3,C4) disynaptic basins integrating 2.43 e, 0.93 e, and 2.92 e, respectively. The population of these basins indicates that while the C1–C2 and C2–C4 double bond have been depopulated, a new V(C1, C3) disynaptic basin is formed, which is related to the creation of a new C1–C3 bond at this TS (Figure 6). ELF of **TS1a-II** also shows the appearance of a new V(C2) monosynaptic basin (0.66 e) connected with the *pseudoradical* C2 center. Similar populations values are found in **TS1b-II**, d(C1,C3) = 1.893 Å and d(C2,C4) = 3.586 Å.

ELF picture of **INa-II**, d(C1,C3) = 1.527 Å and d(C2,C4) = 3.977 Å, is very close to that of **TS1a-II**. A slight growth in the value of V(C1,C3) disynaptic basin and in the decrease in the value of V(C1,C2) and V(C3,C4) disynaptic basins is observed. The population of the V(C2) monosynaptic basin has been increased to 1.02 e. ELF picture of **INb-II**, d(C1,C3) = 1.520 Å and d(C2,C4) = 3.181 Å, is very similar to that of **INa-II**. Only a slight difference in the value of the populations of the valence basins is noticed (Figure 6).

Finally, the ELF picture of **TS2-II**, d(C1,C3) = 1.517 Å and d(C2,C4) = 2.485 Å, shows a great similarity to that of **INb-II**. The only noticeable change is the formation of a new V(C4) monosynaptic basin, integrating 0.38 e, associated with the formation of a new *pseudoradical* C4 center. Note that the C2 and C4 pseudoradical centers are needed for the formation of the second C2–C4 bond.

The present ELF study of the TSs and intermediates indicates that while the creation of the first C1–C3 bond has already begun at **TS1a-II** and **TS1b-II**, the creation of the next C2–C3 bond has not started yet at **TS2-II**.

#### 3.4.3. ELF Study of the TSs and Intermediate Participated in the 22CA Between PFBHE **1** and Benzene **3**

**Figure 7:**
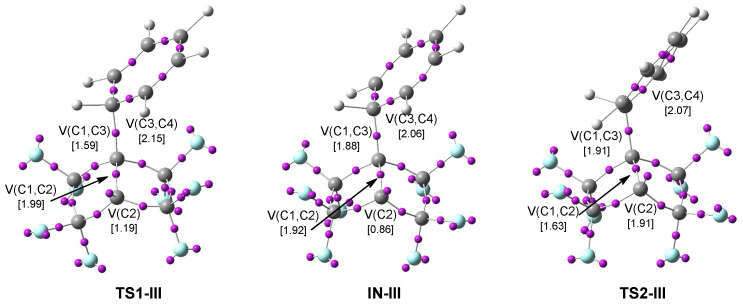
ELF study of the TSs and intermediates participated in the 22CA of the PFBHE **1** and benzene **3**.

The results for the analysis of this part participating in the 22CAs of the PFBHE **1** and benzene **3** are presented in Figure 7. ELF picture of **TS1-III**, d(C1,C3) = 1.631 Å and d(C2,C4) = 3.202 Å, shows the presence of three V(C1,C2), V(C1,C3) and V(C3,C4) disynaptic basins (1.99 e, 1.59 e and 2.15 e, properly). The population of these basins indicates that while the C1–C2 and C3–C4 double bond have been depopulated, a new V(C1,C3) disynaptic basin is established, which is related to the creation of a C1–C3 bond at TS. ELF picture of **TS1-III**, also presents the presence of a new V(C2) monosynaptic basins (1.19 e.) related to the creation of a *pseudoradical* C2 center (Figure 7).

ELF picture of **IN-III**, d(C1,C3) = 1.549 Å and d(C2,C4) = 3.201 Å, does not shows significant changes with respect to that of **TS1-III**, as a result of the very advanced character of the former.

Finally, ELF picture of **TS2-III**, d(C1,C3) = 1.527 Å and d(C2,C4) = 2.598 Å, is very similar to that of **IN-III**. While the population of V(C2) monosynaptic and V(C2,C3) disynaptic basins has increased both to 1.91 e, that of the V(C1,C2) disynaptic basin has been depopulated to 1.63 e.

The present ELF topological analysis of the TSs and intermediates concerned in the 22CA of the PFBHE **1** and benzene **3** states that while the creation of the C1–C3 bond has already begun at **TS1-III**, the creation of the next C2–C3 bond has not started yet at **TS2-III**. In addition, the great similarity between the ELF image of the very unfavorable **TS1-III**, and that of **IN-III**, indicates the very advanced character of the former.

#### 3.4.4. ELF Study of the TSs and Intermediate Participated in the 22CA of the PFBHE **1** and Styrene **12**

**Figure 8:**
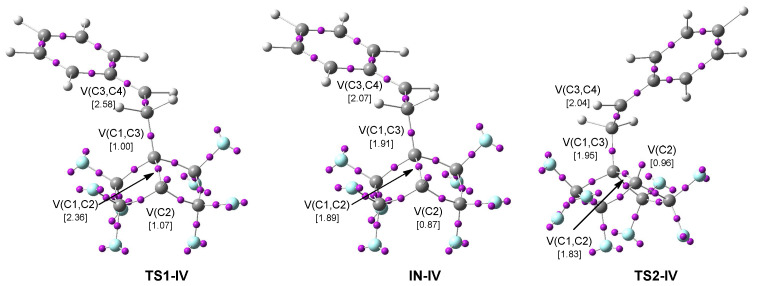
ELF basin attractor positions of the TSs and intermediates in the 22CA of the PFBHE **1** and styrene **12**.

The results for this part of the TSs and intermediates participated in the 22CA of the PFBHE **1** and styrene **12** are summarized in Figure 8. ELF picture of **TS1-IV**, d(C1,C3) = 1.825 Å and d(C2,C4) = 3.896 Å, shows the presence of three V(C1,C2), V(C1,C3), and V(C3,C4) disynaptic basins, integrating 2.36 e, 1.00 e, and 2.58 e (Figure 8). The population of these basins indicates that while the C1–C2 and C3–C4 double bond have been depopulated, a new V(C1,C3) disynaptic basin is formed, which is related to the creation of a new C1–C3 at TS. The ELF picture of **TS1-IV** also presents the presence of one V(C2) monosynaptic basin, integrating 1.07 e, which is associated with the formation of a *pseudoradical* C2 center.

ELF picture of **IN-IV**, d(C1,C3) = 1.526 Å and d(C2,C4) = 3.979 Å, shows only slight changes in the basin populations with respect to those of **TS-IV**; a reduction in the population of the V(C2) monosynaptic and the V(C1,C2) and V(C3,C4) disynaptic basins is observed. In turn, the population of V(C1,C3) disynaptic basin has been increased to 1.91 e.

Finally, the ELF picture of **TS2-IV**, d(C1,C3) = 1.520 Å and d(C2,C4) = 2.717 Å, is very similar to that of **IN-VI**. The population of the V(C2) monosynaptic basis has been increased to reach 0.96 e.

The present ELF study of the TSs and intermediates participated in the 22CA of the PFBHE **1** and styrene **12** indicates that while the creation of the C1–C3 bond has already begun at **TS1-IV**, the creation of the next C2–C3 bond has not started yet at **TS2-IV**.

### 3.5. RIAE Analysis of the Electronic Effects Participating in the Activation Energies Related to the Thermal 22CA Reactions of BHE ***2*** with Ethylene ***17***, and Those of PFBHE ***1*** with Ethylene ***17*** and Styrene ***12***

Finally, in order to get to know the electronic factors responsible for the accelerations found in polar thermal 22CA reactions, an RIAE analysis [17] of **TS-1a-II** and **TS-1a-IV** connected with the thermal 22CA reactions of PFBHE **1** with ethylene **17** and styrene **12**, was carried out. **TS-1a-I** associated with the non-polar thermal 22CA reactions of BHE **2** with ethylene **17** was chosen as the reference. This RIAE analysis was performed at the UM06-2X/6-311+G(d,p) computational level by the IQA calculations [16]. The atoms at the three TSs were regrouped into two frameworks, **A** and **B**, in this RIAE analysis; **A** corresponds with the PFBHE and BHE frameworks, and **B** with the ethylene and styrene ones.

The UM06-2X/6-311+G(d,p) gas-phase ξ$E_{intra}^{X}$ intra-atomic, ξ$E_{inter}^{X}$ interatomic and ξ$E_{total}^{X}$ total energies of the **A** and **B** frameworks, together with the ξ$E_{total}^{A+B}$ RIAE activation energies of the three TSs are provided in Table 5.

RIAE analysis of **TS1a-I**, corresponding with the 22CA reaction of BHE **2** with ethylene **17**, indicates that the initial contributor to the high RIAE activation energy, ξ$E_{total}^{A+B}$ = 27.3 kcal·mol^−1^, is the destabilization of the ethylene **B** framework, ξ$E_{total}^{B}$ = 17.6 kcal·mol^−1^. The BHE **A** structure is destabilized by 9.7 kcal·mol^−1^. Analysis of the unfavorable contribution to the ξ$E_{total}^{B}$ total energy of the framework **B** shows that the unfavorable intra-atomic energies of the ethylene framework, ξ$E_{intra}^{B}=\;$23.0 kcal·mol^−1^, are the primary factors responsible for the height of the high RIAE activation energy.

RIAE analysis of **TS1a-II**, corresponding to the 22CA reaction of PFBHE **1** with ethylene **17**, reveals an interesting finding. While the ethylene **B** framework is strongly destabilized by ξ$E_{total}^{B}$ = 48.3 kcal·mol^−1^, the PFBHE **A** structure is intensively stabilized by ξ$E_{total}^{A}$ = −26.5 kcal·mol^−1^. As a result, the RIAE activation energy of this 22CA reaction decreases to ξ$E_{total}^{A+B}\;$= 21.9 kcal·mol^−1^. A specific study of the electronic factors responsible for this decrease states that the strong, favorable intra-atomic interactions at the PFBHE **A** structure, ξ$E_{intra}^{A}$ = −116.3 kcal·mol^−1^, are responsible for this decrease.

RIAE analysis of **TS1a-IV**, corresponding to the 22CA reaction of PFBHE **1** with styrene **12**, reveals a similar trend to that of the reaction before 22CA. While the ethylene **B** framework is strongly destabilized by ξ$E_{total}^{B}$ = 54.5 kcal·mol^−1^, now the PFBHE **A** framework is more strongly stabilized by ξ$E_{total}^{A}$ = −40.2 kcal·mol^−1^. As a result, the RIAE barrier of this 22CA experience a markedly decrease to ξ$E_{total}^{A+B}$ = 14.3 kcal·mol^−1^. As in the 22CA of PFBHE **1** with ethylene **17**, a detailed study of the electronic factors responsible for this strong decrease indicates that, similar to **TS1a-II**, the strong intra-atomic interactions at the PFBHE **A** framework, ξ$E_{intra}^{A}$ = −160.1 kcal·mol^−1^, are responsible for this strong decrease.

**Figure 9:**
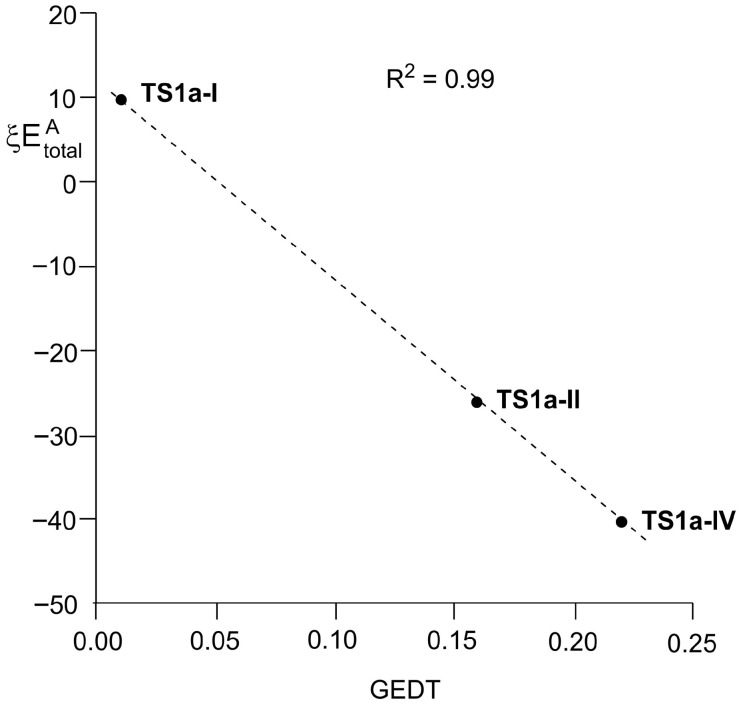
Plot of the ξ$E_{total}^{A}$ total energies of the PFBHE and BHE frameworks, ξ*E* in kcal·mol^−1^, against GEDT at the analogous TSs.

While the 22CA reaction of BHE **2** with ethylene **17** is classified as NEDF, those of PFBHE **1** with ethylene **17** and styrene **12** are classified a REDF. This useful classification of the organic process on the basis of the GEDT at the TSs allows the ubiquitously classified compound **A** PFBHE **1** as an electrophilic species, and the compounds **B** ethylene **17** and styrene **12** as the nucleophilic species. Recent MEDT studies have shown that in a polar reaction, while the nucleophile is destabilized by the loss of electron density, the electrophile is stabilized to gain electron density [17,39]. Thus, as shown in Figure 9, a strong linear correlation between the ξ$E_{total}^{A}$ total energies of the BHE and PFBHE frameworks and the GEDT computed at the corresponding TSs are found, R^2^ = 0.99. As can be seen, the non-polar 22CA reaction of BHE **2**, a marginal electrophile, with ethylene **17**, a marginal nucleophile (see Table 1), is positioned at the top of the graph. In contrast, the polar 22CA reaction of PFBHE **1**, a strong electrophile, with styrene **12**, a strong nucleophile, is located at the bottom (see Figure 9).

**Figure 10:**
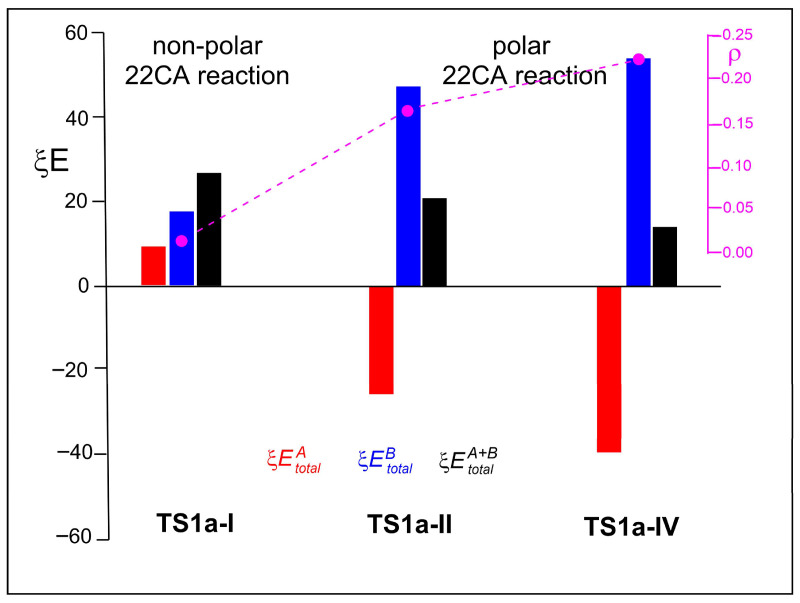
Graphical representation of the total energies, ξ$E_{total}^{A}$, ξ$E_{total}^{B}$, and ξ$E_{total}^{A+B}$ for the TSs given in Table 5 Energies are presented in kcal·mol^−1^ and GEDT in number of electrons e.

Finally, Figure 10 displays a graphical image of the ξ$E_{total}^{X}$ energies of the **A** and **B** structures at the three TSs. The ξ$E_{total}^{A+B}$ energies, show the RIAE relative energies of these A–E reactions.

Some appealing conclusions can be obtained from Figure 10: (i) at the non-polar **TS1a-I** both A and B frameworks are destabilized causing the most RIAE activation energy; (ii) at the two polar 22CA reactions, while the ethylene and styrene nucleophilic frameworks are strongly destabilized, the BHE and PFBHE electrophilic frameworks are strongly stabilized; and (iii) as the stabilizing factors, resulting from the GEDT, are greater that the destabilizing ones, this phenomena causes a reduction in the RIAE activation energies.

The presence of the eight fluorine atoms in PFBHE **1** causes a marked increase in its electrophilic character, as shown by its high electrophilicity ω index, with respect to the hydrocarbon BHE **2**. This behavior causes the existence of a GEDT at the corresponding TSs, which is manifested even in the 22CA reaction with ethylene **17**. As a result of the accumulation of electron density at the electrophilic PFBHE framework, it is strongly electronically stabilized. In spite of the destabilizing of the nucleophilic styrene framework, the stabilization of the electrophilic PFBHE framework is greater, and consequently, a marked stabilization of **TS1a-IV** is produced.

## 4. Conclusions

Thermal 22CA reactions of PFBHE **1** and BHE **2** with ethylene **17**, benzene **3**, and styrene **12** have been examined within the MEDT at the UM06-2X/6-311G(d,p) level in benzene. Study of the DFT-based reactive indices indicates that the presence of the eight fluorines in PFBHE **1** increases the electrophilic nature of this species, allowing its participation in polar 22CA reactions. On the other hand, styrene **12** shows a strong nucleophilic nature.

The studied 22CA reactions undergo via a stepwise mechanism with an intermediate. Due to that the first step of these 22CA reactions is only related to the creation of the C1–C3 bond, two stereoisomeric reaction paths characterized by the different C2–C1–C3–C4 dihedral angle are found. While the reaction paths b directly yield the final [2+2] cycloadduct by a ring-closure at the corresponding intermediate, along the reaction paths a, the corresponding intermediate should be converted into the intermediate b through a C1–C3 single bond rotation.

While the non-polar 22CA of the BHE **2** with ethylene **17**, classified as NEDF, states a high activation energy of 26.6 kcal·mol^−1^, the polar 22CA reaction of PFBHE **1** with styrene **12**, classified as REDF, presents a low activation energy of 13.2 kcal·mol^−1^. The polar 22CA of the PFBHE **1** with benzene **3** presents a high activation energy of 35.9 kcal·mol^−1^, because of the loss of the aromatic nature of benzene **3** along the 22CA reaction. Thus, while the reaction with styrene **12** is strongly exothermic by 45.1 kcal·mol^−1^, that with benzene **3** is exothermic by only 10.0 kcal·mol^−1^. Based on this, it can be concluded that the 22CA reaction between PFBHE **1** and benzene **3** is a non-competitive alternative to the 42CA process.

The analysis of the geometrical parameter together with the ELF of TSs and intermediates associated with the four studied 22CA reaction shows a great similarity between them. Consequently, the substitution in the two interacting ethylenes mainly affects the energetic parameters. ELF study of the TSs connected with the first step indicates that the creation of the C1–C3 bond has begun at all TSs. In contrast, analysis of the TSs connected with the ring-closure reactions shows that formation of the next C2–C4 bond has not begun at any of them.

At the end, an RIAE analysis for the activation energies of these 22CA reactions shows that while at the non-polar **TS1a-I,** both frameworks are electronically destabilized, causing a high RIAE activation energy, at **TS1a-IV**, the high GEDT taking place at the PFBHE **1** framework causes a strong electronic stabilization of this framework, reducing the RIAE activation energy markedly. A strong interdependence between the ξ$E_{total}^{A}$ total energies of the BHE and PFBHE frameworks and GEDT computed at the corresponding TSs are found, R^2^ = 0.99, revealing that, as in [4+2] and [3+2] cycloaddition reactions, the GEDT performs a relevant role in the reduction in the activation energies.

## Figures and Tables

**Table 1:** The reactivity indices at the B3LYP/6-31G(d) level for PFBHE **1**, BHE **2**, ethylene **17**, benzene **3**, and styrene **12**. The values are in eV.

	µ	η	ω	*N*
**1**	−5.38	6.96	2.08	0.26
**12**	−3.43	5.20	1.13	3.09
**3**	−3.30	6.80	0.80	2.42
**17**	−3.37	7.76	0.73	1.87
**2**	−2.46	7.41	0.41	2.95

**Table 2:** The local electronic qualities for PFBHE **1**, BHE **2**, and styrene **12**.

		$P_{k}^{+}$ [eV]	ω_k_ [eV]	$P_{k}^{-}$ [eV]	*N*_k_ [eV]
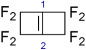 **1**	**C1**	0.48	1.00	0.02	0.01
	**C2**	0.48	1.00	0.02	0.01
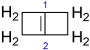 **2**	**C1**	0.49	0.20	0.45	1.33
	**C2**	0.49	0.20	0.45	1.33
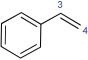 **12**	**C3**	0.01	0.01	0.03	0.09
	**C4**	0.47	0.53	0.50	1.54

**Table 3:** Key geometrical criteria, in Angstroms Å, for stationary points taking part in the 22CA PFBHE **1** (I) and BHE **2** (II) with ethylene **17**. Also, the GEDT at TSs and INs was given.

Structure	Interatomic Distance				GEDT [e]
	C1–C2	C3–C4	C1–C3	C2–C4	
**1**	1.326				
**17**		1.325			
**TS1a-I**			1.829	3.899	0.16
**TS1b-I**			1.846	3.412	0.15
**INa-I**			1.529	3.980	0.23
**INb-I**			1.529	3.101	0.24
**TS2-I**			1.516	2.693	0.20
**18**			1.546	1.546	
**2**	1.313				
**TS1a-II**			1.908	3.997	0.01
**TS1b-II**			1.893	3.586	0.03
**INa-II**			1.527	3.977	0.04
**INb-II**			1.520	3.181	0.03
**TS2-II**			1.517	2.485	0.00
**19**			1.546	1.546	

**Table 4:** Key geometrical criteria, in Angstroms Å, for points connected with the 22CA PFBHE **1** with benzene **3** and styrene **12**. The GEDT at TSs and INs is also given.

Structure	Interatomic Distances				GEDT
	C1–C2	C3–C4	C1–C3	C2–C4	
**1**	1.326				
**3**		1.391			
**TS1-III**			1.631	3.202	0.29
**IN-III**			1.549	3.203	0.33
**TS2-III**			1.527	2.598	0.35
**18**			1.557	1.562	
**12**		1.331			
**TS1a-IV**			1.825	3.896	0.22
**INa-IV**			1.526	3.979	0.34
**TS2-IV**			1.520	2.717	0.28
**19**			1.543	1.567	

**Table 5:** UM06-2X/6-311G(d,p) ξ$E_{total}^{X}$ total, ξ$E_{intra}^{X}$ intra-atomic and ξ$E_{inter}^{X}$ interatomic energies, in kcal mol^−1^, of the **A** and **B** ethylene frameworks at the TSs. The sum of the ξ$E_{total}^{X}$ energies of the two frameworks, i.e., ξ$E_{total}^{A+B}$, yields the RIAE activation energies.

	ƒ(X)	$\xi E_{intra}^{X}$	$\xi E_{inter}^{X}$	$\xi E_{total}^{X}$	$\xi E_{total}^{A+B}$
**TS1a-I**	**A** BHE	9.9	−0.2	9.7	27.3
	**B** ethylene	23.0	−5.4	17.6	
**TS1a-II**	**A** PFBHE	−116.3	89.9	−26.5	21.9
	**B** ethylene	35.5	12.9	48.3	
**TS1a-IV**	**A** PFBHE	−160.1	120.0	−40.2	14.3
	**B** styrene	42.4	12.1	54.5	

## Data Availability

The original contributions presented in this study are included in the article/Supplementary Materials. Further inquiries can be directed to the corresponding authors.

## Supplementary Materials

The following supporting information can be downloaded at https://www.mdpi.com/article/10.3390/ma18245675/s1, Table S1. M06-2X/6-311G(d,p) total energies in benzene, in a.u., of the stationary points involved in the 22CA reaction of PFBHE **1** (I) and BHE **2** (II) with ethylene **17**; Table S2. M06-2X/6-311G(d,p) total energies in benzene, in a.u., of the 22CA reaction of PFBHE **1** with benzene **3** and styrene **12**; M06-2X/6-311G(d,p) computed total energies in benzene, single imaginary frequency, and Cartesian coordinates of the stationary points involved in the 22CA reaction of PFBHE **1** with ethylene **17**; M06-2X/6-311G(d,p) computed total energies in benzene, single imaginary frequency, and Cartesian coordinates of the stationary points involved in the 22CA reaction of BHE **2** with ethylene **17**; M06-2X/6-311G(d,p) computed total energies in benzene, single imaginary frequency, and Cartesian coordinates of the stationary points involved in the 22CA reaction of PFBHE **1** with benzene **3**; M06-2X/6-311G(d,p) computed total energies in benzene, single imaginary frequency, and Cartesian coordinates of the stationary points involved in the 22CA reaction of PFBHE **1** with styrene **12**. References [40,41,42,43,44,45,46,47,48,49,50,51,52,53,54,55,56,57,58,59] are cited in the Supplementary Materials.
