# Supplementary material for: Thermal [2+2] Cycloaddition Reactions of Perfluorobicyclo[2.2.0]hex-1(4)-ene with Ethylene, Benzene and Styrene: A MEDT Perspective
Source: Materials (Basel). 2025 Dec 17;18(24):5675. doi: 10.3390/ma18245675 (PMC12735340; doi:10.3390/ma18245675)
Supplement: Supplementary file 1 [file materials-18-05675-s001.zip › materials-3973908-supplementary.pdf]

Supplementary Material

Thermal [2+2] Cycloaddition Reactions of Perfluorobicyclo[2.2.0]hex-1(4)-ene with Ethylene, Benzene and Styrene: A MEDT Perspective

Agnieszka Kącka-Zych [1,*] and Luis R. Domingo [2,*]

[1] Department of Organic Chemistry and Technology, Faculty of Chemical Engineering and Technology,
Cracow University of Technology, Warszawska 24, 31-155 Cracow, Poland
[2] Independent Researcher, Avd. Tirso de Molina 20, 46015 Valencia, Spain
* Correspondence: agnieszka.kacka-zych@pk.edu.pl (A.K.-Z.); luisrdomingo@gmail.com (L.R.D.)

Index

S3 Theoretical background of the Relative Interacting Atomic Energy (RIAE) Analysis.

S4 Computational Details.

S7 Table with M06-2X/6-311G(d,p) total energies in benzene of the stationary points involved in the 22CA reaction of PFBHE **1** and BHE **2** with ethylene **17**.

S7 Table with the M06-2X/6-311G(d,p) total energies in benzene of the 22CA reaction of PFBHE **1** with benzene **3** and styrene **12**.

S8 M06-2X/6-311G(d,p) computed total energies in benzene, single imaginary frequency, and Cartesian coordinates of the stationary points involved in the 22CA reaction of PFBHE **1** with ethylene **17**.

S13 M06-2X/6-311G(d,p) computed total energies in benzene, single imaginary frequency, and Cartesian coordinates of the stationary points involved in the 22CA reaction of BHE **2** with ethylene **17**.

S18 M06-2X/6-311G(d,p) computed total energies in benzene, single imaginary frequency, and Cartesian coordinates of the stationary points involved in the 22CA reaction of PFBHE **1** with benzene **3**.

S21 M06-2X/6-311G(d,p) computed total energies in benzene, single imaginary frequency, and Cartesian coordinates of the stationary points involved in the 22CA reaction of PFBHE **1** with and styrene **12**.

Theoretical background of the Relative Interacting Atomic Energy (RIAE) Analysis.

The Interacting Quantum Atoms[1] (IQA), based on the Quantum Theory of Atoms in Molecules[2] (QTAIM), divides the E_{total}^{IQA} total energy into two main energy contributions: the E_{intra}^{A} intra-atomic energies and the E_{inter}^{AB} interatomic energies (see Equation S1). The E_{inter}^{AB} energies are, in turn, divided into four additional electrostatic terms: the V_{ne}^{AB} and V_{en}^{AB} nuclei-electron interactions, the V_{ee}^{AB} electron-electron interactions, and the V_{nn}^{AB} nuclei-nuclei interactions (see Equation S3).

$$E_{total}^{IQA} = \sum E_{intra}^{A} + \sum E_{inter}^{AB} \quad \text{(S1)}$$

$$E_{intra}^{A} = T(A) + V_{ne}^{A} + V_{ee}^{A} \quad \text{(S2)}$$

$$E_{inter}^{AB} = ½V_{ne}^{AB} + ½V_{en}^{AB} + ½V_{ee}^{AB} + V_{nn}^{AB} \quad \text{(S3)}$$

Thanks to the additivity of the topological atoms,[3] an Interacting Quantum Fragment (IQF) approach has been recently introduced,[4] which allows the grouping of the IQA energy in terms of convenient fragments of the system. This enables a more chemically meaningful energy analysis of the interactions that take place between the atoms forming groups. In this sense, in the so-called Relative Interacting Atomic Energy[5] (RIAE) analysis, the atoms belonging to the TSs are regrouped in the two frameworks f(X) interacting in the present [2+2] cycloaddition reactions; i.e., the two ethylenes.[6,7]

By default, the sum of all IQA atomic energies belonging to the considered framework f(X) (where X represents either ethylene frameworks) at the TSs, and those of the separated reagents at the ground states (GSs), is computed. The RIAEs, i.e., the relative ξE_{total}^{X} total, ξE_{intra}^{X} intra-atomic, and ξE_{inter}^{X}interatomic energies are obtained using Equations S4-S6. The symbol ξ denotes the IQA energy differences between the GS and the TS states of the two-interacting frameworks f(X); i.e., f(ethilene1) and f(ethylene2) in [2+2] cycloaddition reactions.

$$\xi E_{total}^{X} = \xi E_{intra}^{X} + \xi E_{inter}^{X} \quad \text{(S4)}$$

$$\xi E_{intra}^{X} = \sum E_{intra}^{X(TS)} - \sum E_{intra}^{X(GS)} \quad \text{(S5)}$$

$$\xi E_{inter}^{X} = \sum E_{inter}^{X(TS)} - \sum E_{inter}^{X(GS)} \quad \text{(S6)}$$

The proposed RIAE analysis provides a measure of how much the two interacting frameworks f(X) are destabilized (resulting in positive relative energies) or stabilized (resulting in negative relative energies) when going from their GS to the TS. The sum of the ξE_{total}^{X} energies of the two interacting frameworks, $\xi E_{total}^{2MBD+CHO}$, provides the RIAE

activation energy of [2+2] cycloaddition reactions obtained through the present energy decomposition analysis.[5-7]

Computational Details

This MEDT study has been carried out by performing quantum chemical calculations within DFT.[8] The M06-2X functional,[9] together with the standard 6-311G(d,p)[10] basis set, which includes d-type polarization for second-row elements and p-type polarization functions for hydrogens, were used throughout this MEDT study. The unrestricted formalism[10] UM06-2X was used in all calculations. The TSs were characterized by the presence of only one imaginary frequency. The Berny method was used in optimizations.[11,12] The intrinsic reaction coordinate (IRC)[13] calculations were performed to establish the unique connection given between the TSs and the corresponding minima.[14,15] Solvent effects of benzene were considered by full optimization of the gas phase structures at the same computational level using the polarizable continuum model (PCM)[16,17] in the framework of the self-consistent reaction field (SCRF).[18-20]

The global electron density transfer (GEDT)[21] was computed using the equation GEDT(f) = Σqf, where q are the natural charges[22,23] of the atoms belonging to one of the two frameworks (f) at the TS geometries. Global and local DFT-based reactivity indices[24-28] were calculated using the equations given in reference [25].

The Gaussian 16 suite of programs was used to perform the calculation.[27] Electron localization function (ELF)[28] analyses of the UM06-2X/6-311G(d,p) monodeterminantal wavefunctions were performed by using the TopMod[29] package with a cubical grid of step size of 0.1 Bohr. The IQA analysis was performed using the AIMAll package[30] and the corresponding UM06-2X/6-311G(d,p) monodeterminantal wavefunctions. Molecular geometries and ELF basin attractors were visualized by using the GaussView program.[31]

References

1. Blanco, M.A.; Pendás, M.; Francisco, E. *J. Chem. Theory Comput.,* 2005, *1*, 1096–1109.
2. (a) Bader, R.F.W., Tang, Y.H., Tal, Y. and Biegler-König, F.W. *J. Am. Chem. Soc.*, **1982**, *104*, 946–952; (b) Bader, R.F.W. *Atoms in Molecules: A Quantum Theory*, Oxford University Press, Oxford, New York, **1994**.

3. Pendás, A.M., Blanco, M.A. and Francisco, E. *J. Comput. Chem.,* **2007**, *28*, 161–184.
4. Triestram, L., Falcioni, F. and Popelier, P.L.A, *ACS Omega*, **2023**, *8*, 34844–34851.
5. Domingo, L.R., Ríos-Gutiérrez, M. and Pérez, P. *J. Org. Chem.*, **2024**, *89,* 12349–12359.
6. Domingo, L.R., Pérez, P., Ríos-Gutiérrez, M. and Aurell, M.J. *Tetrahedron Chem.*, **2024**, *10*,100064.
7. Domingo, L.R. and Ríos-Gutiérrez, M. *Molecules*, **2024**, *29*, 1.
8. Zhao, Y.; Truhlar, D.G. The M06 suite of density functionals for main group thermochemistry, thermochemical kinetics, noncovalent interactions, excited states, and transition elements: two new functionals and systematic testing of four M06-class functionals and 12 other functionals, *Theor. Chem. Acc.* **2008**, *120*, 215-241.
9. Hehre, M.J.; Radom, L.; Schleyer, P.Y.R.; People, J. In Ab initio Molecular Orbital Theory, Wiley, New York, **1986**.
10. Schlegel, H.B. Optimization of equilibrium geometries and transition structures, *J. Comput. Chem*., **1982**, *3*, 214-218.
11. Schlegel, H.B. in *Modern Electronic Structure Theory*, ed. D. R Yarkony, World Scientific Publishing: Singapore, **1994**.
12. Fukui, K. Formulation of the reaction coordinate*, J. Phys. Chem*., **1970**, *74*, 4161–4163.
13. González, C.; Schlegel, H.B. Reaction path following in mass-weighted internal coordinates, *J. Phys. Chem*., **1990**, *94*, 5523-5527.
14. González, C.; Schlegel, H.B. Improved algorithms for reaction path following: Higher-order implicit algorithms., *J. Chem. Phys*., **1991**, *95*, 5853-5860.
15. Tomasi, J.; Persico, M. Molecular interactions in solution: and overview of methods based on continuous distributions of the solvent, *Chem. Rev*., **1994**, *94*, 2027-2094.
16. Simkin, B.I.; Sheikhet, I.I. *Quantum chemical and statistical theory of solutions–computational approach*, Ellis Horwood: London, **1995**.
17. Cossi, M.; Barone, V.; Cammi, R.; Tomasi, J. Ab initio study of solvated molecules: A new implementation of the polarizable continuum model, *Chem. Phys. Lett*., **1996**, *255*, 327-335.
18. Cances, E.; Mennucci, B.; Tomasi, J. A new integral equation formalism for the polarizable continuum model: Theoretical background and applications to isotropic and anisotropic dielectrics, *J. Chem. Phys*., **1997**, *107*, 3032-3041.
19. Barone, V.; Cossi, M.; Tomasi, J. Geometry optimization of molecular structures in solution by the polarizable continuum model, *J. Comput. Chem*., **1998**, *19*, 404-417.
20. Domingo, L.R. A New C-C Bond Formation Model Based on the Quantum Chemical Topology of Electron Density, *RSC Adv*., **2014***, 4*, 32415-32428.
21. Reed, A.E.; Weinstock, R.B.; Weinhold, F. Natural population analysis. *J. Chem. Phys*., **1985**, *83*, 735-746.

22. Reed, A.E.; Curtiss, L.A.; Weinhold, F. Intermolecular interactions from a natural bond orbital, donor-acceptor viewpoint. *Chem. Rev.*, **1988**, *88*, 899-926.
23. Parr, R.G.; Yang, W. in *Density Functional Theory of Atoms and Molecules*, Oxford, University Press, New York, **1989**.
24. Domingo, L.R.; Ríos-Gutiérrez, M.; Pérez, P. Applications of the Conceptual Density Functional Theory Indices to Organic Chemistry Reactivity, *Molecules* **2016**, *21,* 748.
25. Sadowski, M.; Kula, K. Unexpected course of reaction between (1E,3E)-1,4-dinitro-1,3-butadiene and N-methyl azomethine ylide - A comprehensive experimental and quantum-chemical study. *Molecules* **2024**, *29(21)*, e5066.
26. Sadowski, M.; Synkiewicz-Misualska, B.; Kula, K. (1E,3E)-1,4-dinitro-1,3-butadiene - Synthesis, spectral characteristics and computational study based on MEDT, ADME and PASS simulation. *Molecules* **2024**, *29(2)*, e542.
27. Frisch, M.J. et al. *Gaussian 16* Gaussian, Inc., Wallingford CT, **2016**.
28. Becke, A.D.; Edgecombe, K.E. A simple measure of electron localization in atomic and molecular systems, *J. Chem. Phys.,* **1990**, *92,* 5397–5403.
29. Noury, S.; Krokidis, X.; Fuster, F.; Silvi, B. Computational tools for the electron localization function topological analysis, *Comput. Chem*., **1999**, *23*, 597-604.
30. AIMAll (Version 19.10.12), Keith, T.A. TK Gristmill Software, Overland Park KS, USA, **2019** (aim.tkgristmill.com).
31. Dennington, R.; Keith, T.A.; Millam, J.M. *GaussView*, Version 6.1, Semichem Inc., Shawnee Mission, KS**, 2016**.

Table S1. M06-2X/6-311G(d,p) total energies in benzene, in a.u., of the stationary points involved in the 22CA reaction of PFBHE **1** (**I**) and BHE **2** (**II**) with ethylene **17**.

PFBHE **1**	-1027.189219	BHE **2**	-233.261476
ethylene **17**	-78.563042		
**TS1a-I**	-1105.717701	**TS1a-II**	-311.780922
**TS1b-I**	-1105.715205	**TS1b-II**	-311.782103
**INa-I**	-1105.734559	**INa-II**	-311.809814
**TSrot-I**	-1105.725456	**TSrot-II**	-311.803202
**INb-I**	-1105.731757	**INb-II**	-311.811430
**TS2-I**	-1105.731701	**TS2-II**	-311.802145
**18**	-1105.833258	**19**	-311.893673

Table S2. M06-2X/6-311G(d,p) total energies in benzene, in a.u., of the 22CA reaction of PFBHE **1** with benzene **3** and styrene **12**.

PFBHE **1**	-1027.189219
benzene **3**	-232.197518
**TS1-III**	-1259.341578
**IN-III**	-1259.342726
**TS2-III**	-1259.329587
**20**	-1259.402647
styrene **12**	-309.580638
**TS1a-IV**	-1336.748814
**INa-IV**	-1336.765127
**TSrot-IV**	-1336.755711
**TS2-IV**	-1336.761953
**21**	-1336.841690

M06-2X/6-311G(d,p) computed total energies in benzene, single imaginary frequency, and Cartesian coordinates of the stationary points involved in the 22CA reaction of PFBHE **1** with ethylene **17**.

PFBHE **1**
E(UM062X) = -1027.189219 A.U.

6	0.000000	-0.000000	0.663072
6	0.000000	-0.000000	-0.663072
6	1.517260	0.000102	0.794665
6	1.517260	-0.000102	-0.794665
6	-1.517260	-0.000102	0.794665
6	-1.517260	0.000102	-0.794665
9	-2.070164	-1.083718	1.339906
9	-2.070424	1.083162	1.340292
9	-2.070424	-1.083162	-1.340292
9	-2.070164	1.083718	-1.339906
9	2.070424	1.083162	-1.340292
9	2.070164	-1.083718	-1.339906
9	2.070164	1.083718	1.339906
9	2.070424	-1.083162	1.340292

Ethylene **17**
E(UM062X) = -78.563042 A.U.

6	-0.662489	0.000000	0.000004
6	0.662489	0.000000	-0.000003
1	-1.230575	-0.923540	-0.000011
1	-1.230575	0.923540	0.000022
1	1.230575	0.923540	0.000010
1	1.230575	-0.923540	-0.000024

TS1a-I
E(UM062X) = -1105.717701 A.U.
Imaginary frequency -1016.1502 cm^{-1}

6	0.000027	-0.690654	-0.786987
6	0.000012	0.583168	-0.147477
6	0.000806	2.087241	-1.188756
6	0.000943	3.179781	-0.318084
6	1.409378	-1.021061	-0.376096
6	1.388027	0.311894	0.450574
6	-1.409240	-1.020935	-0.375675
6	-1.388508	0.312481	0.449560
1	-0.927063	3.571345	0.078689
1	-0.911611	1.904459	-1.747544
9	-2.331705	-1.039717	-1.357233
9	-1.577104	-2.120952	0.369913

9	-2.351720	1.208098	0.165482
9	-1.394434	0.097706	1.770924
9	1.392541	0.095716	1.771729
9	2.351574	1.207823	0.168481
9	1.577904	-2.121881	0.368084
9	2.331615	-1.038157	-1.357914
1	0.928891	3.571344	0.078795
1	0.913067	1.903645	-1.747494

TS1b-I
E(UM062X) = -1105.715205 A.U.
Imaginary frequency -978.9561 cm^{-1}

6	-0.117072	-0.419906	-0.842378
6	0.079434	0.644834	0.084880
6	0.469052	2.387718	-0.383350
6	1.707493	2.458493	-1.015570
6	1.154777	-1.128785	-0.472100
6	1.264601	-0.114006	0.711369
6	-1.587174	-0.552525	-0.579070
6	-1.410074	0.565786	0.497387
1	2.612671	2.672190	-0.465003
1	0.387471	2.849049	0.597405
9	-2.421299	-0.230530	-1.587135
9	-1.998694	-1.723434	-0.067814
9	-2.139329	1.679673	0.315511
9	-1.618424	0.132398	1.744795
9	0.976765	-0.688734	1.886958
9	2.428195	0.543377	0.844771
9	1.081031	-2.414075	-0.112933
9	2.161618	-0.998874	-1.364474
1	1.807819	2.201013	-2.062861
1	-0.402943	2.529887	-1.013669

INa-I
E(UM062X) = -1105.734559 A.U.

6	0.101990	-0.695648	-0.963202
6	-0.081048	0.712231	-0.343840
6	-0.059608	2.046701	-1.090001
6	-0.308622	3.178895	-0.152450
1	0.503245	3.595987	0.426061
6	1.496916	-0.833028	-0.406400
6	1.179424	0.387993	0.510181
6	-1.136650	-1.220720	-0.254291
6	-1.428821	0.231815	0.233916
1	0.912234	2.141643	-1.580098
9	-2.077706	-1.735699	-1.052581

9	-0.901532	-2.080923	0.746991
9	-2.461242	0.770162	-0.441499
9	-1.653744	0.386000	1.539256
9	0.937542	0.006083	1.771539
9	2.111454	1.349335	0.520282
9	1.843752	-1.966432	0.200320
9	2.440323	-0.514983	-1.312050
1	-0.825859	2.001293	-1.865626
1	-1.320738	3.489752	0.065863

TSrot-I
E(UM062X) = -1105.725456 A.U.
Imaginary frequency 65.6015 cm^{-1}

6	-2.020260	2.610536	-0.538521
6	-0.603686	2.171590	-0.652617
6	-0.278557	0.742118	-0.129717
6	-1.375178	-0.234575	0.371623
6	0.262899	-0.413219	-1.007089
1	-2.420582	2.895750	0.424615
6	1.679571	-0.207314	-0.543530
6	1.090297	0.622677	0.631201
6	-0.674917	-1.418305	-0.363401
1	0.029888	2.861101	-0.091710
9	-1.467410	-2.085203	-1.209843
9	-0.093872	-2.300639	0.462473
9	-2.571001	0.002766	-0.192644
9	-1.550781	-0.364665	1.687877
9	1.046687	-0.096122	1.762133
9	1.713369	1.777964	0.886526
9	2.423323	-1.265372	-0.228942
9	2.376601	0.587787	-1.377311
1	-0.274329	2.210154	-1.692262
1	-2.708235	2.483289	-1.360742

INb-I
E(UM062X) = -1105.731757 A.U.

6	0.082061	-0.172759	-1.004597
6	-0.130326	0.797990	0.187946
6	-0.288136	2.310637	0.027947
6	-1.133461	2.676518	-1.149387
1	-2.207429	2.576056	-1.111745
6	1.534293	-0.383530	-0.662348
6	1.238809	0.270988	0.722095
6	-1.034099	-1.086807	-0.529220
6	-1.358113	-0.051391	0.593202
1	-0.709825	2.701883	0.959864
9	-2.027776	-1.268822	-1.405968

9	-0.645014	-2.281284	-0.061834
9	-2.511342	0.595780	0.349147
9	-1.410980	-0.535364	1.835786
9	1.163846	-0.651776	1.691109
9	2.104128	1.219386	1.098465
9	2.027843	-1.620218	-0.650995
9	2.345418	0.404813	-1.392371
1	0.708083	2.742091	-0.079292
1	-0.672114	3.047467	-2.052718

TS2-I
E(UM062X) = -1105.731701 A.U.
Imaginary frequency -160.7903 cm^{-1}

6	0.044608	-0.036547	-0.978796
6	-0.061593	0.786786	0.330732
6	-0.096511	2.291216	0.143334
6	-0.686500	2.544347	-1.217000
6	1.476460	-0.417003	-0.685953
6	1.258389	0.098112	0.774955
6	-1.148788	-0.895811	-0.594211
6	-1.366525	0.022888	0.651545
1	-0.260490	3.296717	-1.865547
1	0.920377	2.681583	0.195501
9	-2.165955	-0.884260	-1.464309
9	-0.868550	-2.168335	-0.275878
9	-2.445201	0.813545	0.492879
9	-1.474110	-0.597975	1.827082
9	1.111170	-0.918878	1.635747
9	2.209168	0.919482	1.234469
9	1.857640	-1.686086	-0.826778
9	2.352171	0.375250	-1.333028
1	-1.691717	2.206055	-1.428838
1	-0.672405	2.777038	0.939580

18
E(UM062X) = -1105.833258 A.U.

6	-1.421533	-0.115341	0.721031
6	-0.023169	0.526068	0.781019
6	0.023151	0.527789	-0.779560
6	-1.236653	-0.350613	-0.814990
6	1.236765	-0.352520	0.814215
6	1.421681	-0.113375	-0.721046
6	0.106775	2.066527	0.775909
1	-0.632843	2.592767	1.374428
6	-0.106160	2.068348	-0.771083
1	-1.104738	2.383669	-1.071326

9	2.238850	0.077177	1.589401
9	0.996307	-1.644400	1.086579
9	2.392394	0.790719	-0.955126
9	1.657687	-1.196552	-1.462655
9	-0.997440	-1.641448	-1.091872
9	-2.238895	0.082609	-1.588475
9	-1.657567	-1.200143	1.460055
9	-2.392136	0.788275	0.957157
1	1.105633	2.380653	1.076381
1	0.634024	2.595476	-1.368022

M06-2X/6-311G(d,p) computed total energies in benzene, single imaginary frequency, and Cartesian coordinates of the stationary points involved in the 22CA reaction of BHE **2** with ethylene **17**.

BHE **2**
E(UM062X) = -233.261476 A.U.

6	-0.000000	-0.000000	0.656311
6	0.000000	-0.000000	-0.656311
6	1.521736	0.000029	0.797415
6	1.521736	-0.000029	-0.797415
6	-1.521736	-0.000029	0.797415
6	-1.521736	0.000029	-0.797415
1	-1.967166	0.889690	1.246942
1	1.967056	0.889873	1.246806
1	1.967166	0.889690	-1.246942
1	1.967166	-0.889690	1.246942
1	1.967056	-0.889873	-1.246806
1	-1.967166	-0.889690	-1.246942
1	-1.967056	-0.889873	1.246806
1	-1.967056	0.889873	-1.246806

TS1a-II
E(UM062X) = -311.780922 A.U.
Imaginary frequency -1430.8338 cm^{-1}

6	1.158568	-0.000098	-0.620869
6	-0.047953	0.000068	0.102267
6	-1.733499	0.000076	-0.791647
6	-2.766243	0.000085	0.137100
6	1.541444	1.418123	-0.225939
6	0.264214	1.390362	0.686675
6	1.541038	-1.418440	-0.225997
6	0.264013	-1.390183	0.686891
1	-3.128196	-0.924558	0.570361
1	-1.572771	-0.911090	-1.361770
1	-3.128102	0.924732	0.570432
1	-1.572754	0.911244	-1.361761
1	-0.493811	-2.162000	0.524524
1	0.517329	-1.350420	1.747929
1	2.491498	-1.510413	0.305023
1	0.517221	1.350846	1.747796
1	-0.493389	2.162305	0.523881
1	1.521319	2.156729	-1.031285
1	2.491799	1.509678	0.305346
1	1.520363	-2.157014	-1.031360

TS1b-II

E(UM062X) = -311.782103 A.U.
Imaginary frequency -1393.1978 cm^{-1}

6	0.892225	0.331130	-0.683547
6	0.020661	-0.193191	0.290727
6	-1.651238	-0.991891	-0.095346
6	-2.657749	-0.075925	-0.381218
6	0.668571	1.783582	-0.322503
6	-0.116930	1.214954	0.907336
6	1.951750	-0.744581	-0.536718
6	1.054660	-1.296749	0.621073
1	-3.274600	0.343981	0.403301
1	-1.730157	-1.531617	0.846266
1	-2.784490	0.325473	-1.379345
1	-1.287043	-1.610414	-0.911313
1	0.692188	-2.323963	0.522325
1	1.510073	-1.163788	1.603622
1	2.935096	-0.385859	-0.223828
1	0.446078	1.320559	1.836324
1	-1.147826	1.562282	1.035265
1	0.033556	2.326141	-1.031030
1	1.554602	2.383154	-0.105843
1	2.080826	-1.409917	-1.394565

INa-II
E(UM062X) = -311.809814 A.U.

6	-1.166835	-0.038023	-0.747509
6	0.215013	0.034554	-0.074550
6	1.565892	-0.074733	-0.777982
6	2.698013	0.006063	0.188843
1	3.075475	-0.882830	0.676505
6	-1.502094	-1.381081	-0.138467
6	-0.235159	-1.200966	0.760388
6	-1.616217	1.257695	-0.088160
6	-0.190316	1.395027	0.536010
1	1.595314	-1.021556	-1.327481
1	1.631832	0.733710	-1.514121
1	3.026519	0.965750	0.566283
1	0.465439	-2.038339	0.743479
1	-0.473777	-0.952014	1.795740
1	-1.382247	-2.212732	-0.836084
1	-2.467961	-1.469851	0.362812
1	-2.440072	1.143049	0.621944
1	-0.149739	1.451215	1.625304
1	0.375992	2.227619	0.113292
1	-1.866562	2.064763	-0.779123

TSrot-II
E(UM062X) = -311.803202 A.U.
Imaginary frequency -117.9943 cm^{-1}

6	-2.723467	0.028047	0.013046
6	-1.540278	-0.713388	-0.504221
6	-0.158558	-0.271942	0.050193
6	-0.057800	1.079598	0.826516
6	1.001298	0.275547	-0.802954
1	-3.222004	0.782704	-0.579293
6	0.879059	1.681680	-0.268122
6	1.990215	-0.710819	-0.199369
6	0.805665	-1.360780	0.578957
1	-1.504591	-0.635733	-1.593623
1	-1.655009	-1.778746	-0.271999
1	-3.057421	-0.114316	1.032692
1	-1.005282	1.603273	0.963907
1	0.439681	0.964845	1.791510
1	0.326860	2.346696	-0.936176
1	1.796004	2.175325	0.059329
1	2.764802	-0.245943	0.417551
1	0.912486	-1.409926	1.664606
1	0.553952	-2.352119	0.197157
1	2.473720	-1.383713	-0.909935

INb-II
E(UM062X) = -311.811430 A.U.

6	0.686292	0.377712	-0.828954
6	-0.121132	-0.357358	0.258862
6	-1.516607	-0.929397	0.070194
6	-2.463562	0.061304	-0.512670
1	-2.150078	0.657066	-1.361231
6	0.548541	1.728756	-0.165524
6	-0.010411	0.965746	1.077605
6	1.908614	-0.496881	-0.589744
6	1.076137	-1.316309	0.446932
1	-1.431633	-1.805431	-0.591488
1	-1.891276	-1.303984	1.029474
1	-3.505938	0.084548	-0.227557
1	-0.965183	1.341639	1.451240
1	0.707296	0.895593	1.896712
1	-0.222794	2.353757	-0.622201
1	1.460589	2.314296	-0.034139
1	2.777337	0.034724	-0.191247
1	1.486467	-1.359077	1.457429
1	0.858381	-2.329747	0.104469
1	2.229595	-1.084827	-1.451658

TS2-II
E(UM062X) = -311.802145 A.U.
Imaginary frequency -354.3714 cm^{-1}

6	-0.391131	0.132674	-0.843109
6	0.118785	-0.158814	0.564584
6	1.538215	-0.690628	0.504654
6	2.083907	-0.075041	-0.761969
6	-1.401908	-1.000830	-0.800091
6	-1.090934	-1.119821	0.735405
6	-0.758110	1.568530	-0.481963
6	-0.138028	1.323109	0.934639
1	2.719106	-0.642275	-1.430610
1	1.520331	-1.779998	0.406931
1	2.180359	1.003773	-0.808855
1	2.139835	-0.455026	1.392476
1	-0.847458	-2.126573	1.079643
1	-1.066221	-1.887510	-1.341955
1	-1.879526	-0.707190	1.367433
1	-2.428078	-0.771344	-1.095730
1	-1.832027	1.776988	-0.498511
1	-0.805111	1.487527	1.782418
1	-0.250097	2.340990	-1.062007
1	0.784110	1.885572	1.095873

19
E(UM062X) = -311.893673 A.U.

6	0.475347	-1.471235	0.775241
6	-0.000250	0.000191	0.766286
6	-0.000159	-0.000063	-0.766263
6	0.687166	-1.385298	-0.774639
6	1.036653	1.147425	0.774790
6	0.856435	1.287379	-0.775150
6	-1.512214	0.324549	0.774721
1	-2.146863	-0.331707	1.373071
6	-1.543403	0.097162	-0.774975
1	-2.019316	-0.843507	-1.060936
1	-1.715758	1.358932	1.060689
1	-1.976577	0.900702	-1.373442
1	-0.317543	-2.165425	1.062492
1	0.281595	2.170520	-1.062720
1	0.784891	2.025404	1.372477
1	0.207184	-2.162251	-1.372491
1	1.361769	-1.691196	1.373013
1	1.739600	-1.327745	-1.061300
1	2.034105	0.806987	1.061726
1	1.769468	1.258620	-1.372648

M06-2X/6-311G(d,p) computed total energies in benzene, single imaginary frequency, and Cartesian coordinates of the stationary points involved in the 22CA reaction of PFBHE **1** with benzene **3**.

Benzene **3**
E(UM062X) = -232.197518 A.U.

6	0.456651	1.314259	-0.000000
6	1.366572	0.261645	0.000000
6	0.909912	-1.052592	-0.000004
6	-0.456665	-1.314254	0.000000
6	-1.366570	-0.261659	0.000003
6	-0.909900	1.052602	0.000000
1	0.812361	2.337733	-0.000000
1	2.430758	0.465477	-0.000002
1	1.618440	-1.872358	-0.000005
1	-0.812340	-2.337740	-0.000000
1	-2.430762	-0.465456	0.000004
1	-1.618456	1.872344	0.000001

TS1-III
E(UM062X) = -1259.341578 A.U.
Imaginary frequency -1356.6177 cm^{-1}

6	-1.062531	0.463305	-0.911616
6	-0.063763	-0.323026	-0.115740
6	1.323113	-0.927467	-0.724082
6	2.078899	0.120485	-1.425888
6	2.068046	-1.627884	0.336384
6	3.335075	0.484277	-1.032882
1	3.856019	1.277146	-1.554878
1	1.580264	-2.463413	0.827166
6	-2.195429	-0.452890	-0.523413
6	-1.157508	-1.374287	0.177918
6	-0.700884	1.754905	-0.227032
6	0.077121	0.904889	0.816468
6	3.951579	-0.152361	0.058494
1	4.933595	0.166967	0.383010
6	3.308914	-1.218980	0.722919
1	3.818185	-1.726323	1.532902
1	1.581494	0.635192	-2.240463
1	0.900858	-1.642877	-1.438273
9	0.132084	2.537896	-0.951866
9	-1.679130	2.525696	0.255371
9	1.328021	1.292451	1.114819
9	-0.614542	0.800072	1.963017
9	-1.402591	-1.669671	1.457308
9	-0.950836	-2.534031	-0.481824
9	-3.089810	0.063583	0.341642

9	-2.883885	-1.032940	-1.522759

IN-III

E(UM062X) = -1259.342726 A.U.

6	-1.044944	0.286398	-0.986501
6	0.029238	-0.244753	-0.016063
6	1.341914	-0.955631	-0.427877
6	2.079966	-0.201974	-1.491735
6	2.167794	-1.225721	0.794927
6	3.367702	0.197077	-1.332985
1	3.861106	0.733805	-2.134920
1	1.701910	-1.791488	1.594359
6	-1.974539	-0.866144	-0.705121
6	-1.088800	-1.185402	0.535315
6	-1.106194	1.623901	-0.268052
6	0.088958	1.162047	0.625078
6	4.075517	-0.060184	-0.134962
1	5.093370	0.285857	-0.018766
6	3.444739	-0.778759	0.910283
1	3.998826	-0.985341	1.818686
1	1.537656	0.019150	-2.405106
1	1.004650	-1.913724	-0.853777
9	-0.842394	2.691454	-1.030126
9	-2.233611	1.853573	0.420254
9	1.215830	1.818248	0.315048
9	-0.099995	1.252572	1.943561
9	-1.673788	-0.778516	1.669294
9	-0.731786	-2.469525	0.675611
9	-3.263082	-0.621616	-0.477943
9	-1.869578	-1.841011	-1.628400

TS2-III

E(UM062X) = -1259.329587 A.U.

Imaginary frequency -103.3741 cm^{-1}

6	-0.547289	-0.121406	-1.019686
6	0.021692	-0.088300	0.470606
6	1.404979	-0.718866	0.613950
6	1.733150	-1.326415	-0.704484
6	2.452572	0.155762	1.194077
6	2.964505	-1.125914	-1.294088
1	3.201453	-1.586337	-2.243558
1	2.221535	0.653985	2.129307
6	-1.558127	-1.148178	-0.582951
6	-1.202902	-0.920850	0.908960
6	-0.980591	1.312790	-0.864446
6	-0.156817	1.434921	0.443466
6	3.904519	-0.335838	-0.645007

1	4.877846	-0.202800	-1.105741
6	3.661852	0.299755	0.602397
1	4.432347	0.915287	1.045991
1	1.023587	-2.027508	-1.119521
1	1.336581	-1.570779	1.325744
9	-0.584437	2.178187	-1.829960
9	-2.297923	1.577903	-0.653303
9	1.020981	2.066998	0.215003
9	-0.733819	2.034406	1.495484
9	-2.145190	-0.234774	1.574311
9	-0.906970	-2.028918	1.628196
9	-2.852172	-1.019020	-0.913950
9	-1.198094	-2.428851	-0.934557

20
E(UM062X) = -1259.402647 A.U.

6	-1.642782	-0.711276	-0.811078
6	-0.243665	-0.058976	-0.837304
6	-0.125027	-0.290635	0.705742
6	-1.610924	-0.698162	0.753286
6	-0.119434	1.460046	-0.606061
6	0.253017	1.194906	0.891134
6	3.346764	-0.365233	-0.721638
1	4.271673	0.077208	-1.072206
6	3.341928	-0.973613	0.618465
1	4.279431	-1.015256	1.160126
6	2.263360	-0.331225	-1.497801
1	2.289646	0.131208	-2.477876
6	2.224373	-1.432335	1.180665
1	2.223214	-1.848274	2.181707
6	0.980124	-0.989980	-1.079104
1	0.737035	-1.790392	-1.780665
6	0.927318	-1.411705	0.430658
1	0.421942	-2.372955	0.549386
9	0.790834	2.118771	-1.328376
9	-1.293207	2.107738	-0.701902
9	1.556268	1.394990	1.124435
9	-0.446037	1.899612	1.786349
9	-2.435345	0.229870	1.259034
9	-1.875556	-1.874253	1.336592
9	-2.660833	-0.072216	-1.387488
9	-1.613151	-1.974780	-1.280005

M06-2X/6-311G(d,p) computed total energies in benzene, single imaginary frequency, and Cartesian coordinates of the stationary points involved in the 22CA reaction of PFBHE **1** with styrene **12**.

Styrene **12**
E(UM062X) = -309.580638 A.U.

6	1.352276	1.326228	-0.000016
6	-0.013785	1.086512	-0.000050
6	-0.510272	-0.222927	-0.000035
6	0.406219	-1.278633	0.000002
6	1.776068	-1.040763	0.000036
6	2.254278	0.263911	0.000029
1	1.718006	2.346274	-0.000028
1	-0.698767	1.925989	-0.000095
1	0.035910	-2.298454	0.000008
1	2.468135	-1.874439	0.000068
1	3.320615	0.455017	0.000051
6	-1.951971	-0.533715	-0.000058
6	-2.957726	0.338269	0.000078
1	-2.187375	-1.595193	-0.000191
1	-3.985055	-0.003518	0.000046
1	-2.801987	1.411039	0.000232

TS1a-IV
E(UM062X) = 1336.748814 A.U.
Imaginary frequency -723.7466 cm^{-1}

6	2.031703	0.390327	-0.690172
6	0.780449	-0.154639	-0.267731
6	-0.428345	-0.638488	-1.546821
6	-1.489372	-1.265227	-0.881729
6	1.750526	1.750486	-0.138658
6	0.410345	1.169286	0.418780
6	2.819508	-0.805096	-0.255454
6	1.485739	-1.383649	0.322902
1	-1.320344	-2.292047	-0.566557
1	0.223174	-1.301354	-2.106131
9	3.365797	-1.571373	-1.228744
9	3.771010	-0.606972	0.673141
9	1.097411	-2.591550	-0.142849
9	1.481358	-1.439971	1.661702
9	0.381374	1.115220	1.756861
9	-0.720984	1.776199	0.002406
9	2.581900	2.200999	0.818363
9	1.582266	2.760387	-1.024813
1	-0.610887	0.315555	-2.030350
6	-2.722502	-0.680075	-0.450683
6	-3.543385	-1.423024	0.422518

6	-3.165053	0.586255	-0.886189
6	-4.751699	-0.912512	0.862232
1	-3.210867	-2.400628	0.753195
6	-4.374887	1.089053	-0.446249
1	-2.560548	1.165868	-1.570948
6	-5.167413	0.345036	0.429403
1	-5.371187	-1.488017	1.538189
1	-4.709944	2.061267	-0.784725
1	-6.114285	0.746511	0.769825

INa-IV
E(UM062X) = -1336.765127 A.U.

6	2.108738	0.384376	-0.859188
6	0.736636	-0.201821	-0.439098
6	-0.457667	-0.446317	-1.356470
6	-1.564591	-1.124776	-0.617353
6	1.818187	1.711297	-0.204770
6	0.627541	1.016388	0.524383
6	2.858357	-0.741530	-0.165367
6	1.488149	-1.431941	0.115325
1	-0.764040	0.504930	-1.791998
9	3.681548	-1.447270	-0.947735
9	3.516022	-0.391481	0.949026
9	1.313637	-2.501662	-0.683965
9	1.258213	-1.806302	1.374081
9	0.918538	0.762256	1.806985
9	-0.535557	1.674647	0.464173
9	2.750166	2.272287	0.562680
9	1.367520	2.625003	-1.084926
1	-0.108770	-1.082078	-2.175500
1	-1.363596	-2.138455	-0.285031
6	-2.838139	-0.581233	-0.321912
6	-3.235504	0.731371	-0.679715
6	-3.784435	-1.385845	0.364297
6	-4.500694	1.197089	-0.370780
1	-2.542457	1.389317	-1.187608
6	-5.044765	-0.910152	0.667590
1	-3.500503	-2.392962	0.649545
6	-5.415096	0.385470	0.301232
1	-4.780743	2.205208	-0.652465
1	-5.747696	-1.546452	1.191898
1	-6.403272	0.758936	0.539231

TSrot-IV
E(UM062X) -1336.755711A.U.
Imaginary frequency -36.8261 cm^{-1}

6	-1.579915	-1.618128	0.244936
6	-0.321123	-1.543828	-0.548699
6	0.803307	-0.588508	-0.048647
6	0.513532	0.527798	1.016097
6	1.404872	0.555816	-0.901233
6	0.776892	1.628016	-0.052335
6	2.823687	0.058914	-0.696313
6	2.237720	-1.189358	0.027331
1	-0.534702	-1.269683	-1.582570
9	3.500526	-0.221900	-1.815901
9	3.594134	0.824145	0.089423
9	2.348485	-2.279764	-0.753025
9	2.749114	-1.481287	1.223184
9	1.433112	0.592762	1.993884
9	-0.695103	0.547889	1.575648
9	1.515047	2.670322	0.324540
9	-0.381240	2.079251	-0.569918
1	0.122525	-2.540153	-0.583204
1	-1.585400	-2.268067	1.112493
6	-2.761323	-0.884703	-0.037011
6	-2.843165	0.073906	-1.074308
6	-3.916507	-1.095013	0.754607
6	-4.017104	0.770158	-1.304883
1	-1.976977	0.285403	-1.689370
6	-5.084933	-0.397887	0.514134
1	-3.871600	-1.820273	1.559785
6	-5.145574	0.539297	-0.519033
1	-4.055443	1.503396	-2.101723
1	-5.956190	-0.578904	1.132319
1	-6.061089	1.086629	-0.706089

TS2-IV
E(UM062X) = -1336.761953 A.U.
Imaginary frequency -177.2858 cm^{-1}

6	0.722516	-0.106739	0.766013
6	0.900978	-0.073998	-0.776447
6	-0.327320	-0.382806	-1.616248
6	-1.306649	-1.112929	-0.734012
6	1.918396	-1.028929	0.933306
6	1.999820	-1.148298	-0.621383
6	0.823191	1.395652	0.863089
6	1.356780	1.402029	-0.606885
1	-1.048316	-2.132518	-0.467566
1	-0.052233	-1.003108	-2.474215
9	-0.387562	1.982900	0.920298
9	1.609240	1.939913	1.791422
9	0.760060	2.283235	-1.419917
9	2.681702	1.600118	-0.651766

9	3.195937	-0.934256	-1.171230
9	1.552106	-2.350028	-1.035482
9	3.008636	-0.468628	1.480069
9	1.672544	-2.185834	1.559681
1	-0.747244	0.539914	-2.013064
6	-2.591803	-0.638142	-0.360194
6	-3.041404	0.677191	-0.626910
6	-3.473348	-1.508054	0.328002
6	-4.304291	1.084993	-0.235697
1	-2.388291	1.387604	-1.117879
6	-4.732656	-1.090941	0.713393
1	-3.143704	-2.517982	0.545824
6	-5.159310	0.208053	0.431622
1	-4.626974	2.097656	-0.445616
1	-5.390311	-1.776037	1.234743
1	-6.146286	0.535196	0.734200

21
E(UM062X) = -1336.841690 A.U.

6	1.749731	-1.279073	0.421532
6	0.436127	-0.481473	0.258644
6	1.097703	0.348569	-0.883482
6	2.459833	-0.285354	-0.557321
6	0.233001	0.816491	1.068043
6	0.659667	1.649269	-0.184615
6	-0.621774	-1.026846	-0.759924
1	-0.530671	-2.110538	-0.830059
6	0.150751	-0.302144	-1.912685
1	0.670286	-0.993279	-2.575024
9	-0.983347	1.065217	1.553238
9	1.130768	0.960138	2.059070
9	-0.402610	2.212055	-0.790220
9	1.575486	2.595999	0.027755
9	3.308417	0.528041	0.088127
9	3.100760	-0.857279	-1.583867
9	2.285166	-1.390667	1.637668
9	1.664217	-2.510343	-0.118699
1	-0.404339	0.410599	-2.517012
6	-2.065944	-0.695451	-0.466173
6	-2.701333	-1.421140	0.545384
6	-2.781815	0.289837	-1.137521
6	-4.020203	-1.161753	0.884822
1	-2.148444	-2.191799	1.073873
6	-4.109522	0.547829	-0.802013
1	-2.317042	0.876428	-1.918978
6	-4.730722	-0.172114	0.208607
1	-4.496493	-1.731475	1.673618
1	-4.654314	1.318602	-1.333747
1	-5.761702	0.033146	0.469897
